# The root cortex of the Poaceae: a diverse, dynamic, and dispensable tissue

**DOI:** 10.1007/s11104-025-07498-0

**Published:** 2025-04-30

**Authors:** Dylan H. Jones, Kaisa Kajala, Dorota Kawa, Ivan Lopez-Valdivia, Tino Kreszies, Hannah M. Schneider

**Affiliations:** 1 Leibniz Institute of Plant Genetics & Crop Plant Research (IPK) OT Gatersleben, Corrensstr 3, 06466 Seeland, Germany; 2 Experimental & Computational Plant Development, Institute of Environmental Biology, Department of Biology, Utrecht University, Padualaan 8, 3584 CH Utrecht, the Netherlands; 3 Plant Stress Resilience, Institute of Environmental Biology, Department of Biology, Utrecht University, Padualaan 8, 3584 CH Utrecht, the Netherlands; 4 Centre for Crop Systems Analysis, Wageningen University & Research, Wageningen, the Netherlands; 5 Division of Root Sciences, Department of Crop Science, Georg-August-University Goettingen, Goettingen, Germany

**Keywords:** Poaceae, Cortex, Root, Anatomy, Tissue, Cell type

## Abstract

**Background:**

The root cortex in Poaceae is a diverse, dynamic, and dispensable composite layer of tissue. Early in plant growth, the cortex in Poaceae roots primarily consists of parenchyma cells. However, as the root continues to develop, while encountering varying environmental conditions, the cortex undergoes substantial structural and functional changes. These modifications involve either cell wall modifications or programmed cell death, to form tissues including the endodermis, exodermis, sclerenchyma, and aerenchyma, or to result in cortical senescence. The dynamic spatial architecture of these tissues plays a crucial role in storage, microbial interactions, physical protection, biosynthesis of metabolites, and the radial movement of water, nutrients, and gases, and therefore, biotic and abiotic stress tolerance.

**Scope:**

Here, we aim to explore cortical tissues in axial roots of Poaceae and how their capacity for plastic responses to environmental cues underscores their role in plant adaptation and climate resilience. We also highlight key research gaps and opportunities to facilitate our understanding of this composite layer of tissue and its role in plant stress response and rhizosphere interactions.

**Conclusions:**

Axial root cortical tissues and their capacity for dynamic change are major drivers of adaptation and resilience in the Poaceae. Understanding the function and variability of root cortical tissues has potential to improve plant stress tolerance to a number of abiotic and biotic factors across a range of species and environments. Cortical tissues, and the plasticity thereof, may be useful breeding targets for improved soil resource capture and stress tolerance.

## Introduction

### Poaceae

The Poaceae are a family of monocotyledonous plants, generally known as grasses. Comprising over 12,000 species distributed across more than 700 genera, Poaceae occupies diverse habitats, from lush tropical rainforests (e.g. *Bambusa vulgaris)*, waterlogged wetlands (e.g. *Oryza sativa*), arid deserts (e.g. *Aristida* spp.), and alpine regions (e.g. *Deschampsia cespitosa*) (Belsare 2007). This family is distinguished by its unique morphology, frequently including hollow stems, narrow leaves arranged in sheaths, and spikelet inflorescences, which contribute to its remarkable adaptability and ecological success (Nelson and Moore 2020). Poaceae includes the cereals, grass grain crops such as wheat (*Triticum aestivum*), maize (*Zea mays*), rice (*Oryza sativa*), sorghum (*Sorghum bicolor*), barley (*Hordeum vulgare*), pearl millet (*Pennisetum glaucum*), rye (*Secale cereale*), and oat (*Avena sativa*). These grains supply over half of all direct human caloric consumption and are vital sources of bioenergy, which makes the Poaceae the most economically and anthropogenically important plant family. Beyond the cereals, the Poaceae include many other groups of anthropogenic significance such as bamboo (*Bambusa vulgaris*), perennial ryegrass (*Lolium perenne*), sugarcane (*Saccharum officinarum*), dune grass (*Ammophila breviligulata*), and stiff brome (*Brachypodium distachyon*; research model organism).

Further to the specific varieties that have found human applications, grasses are of great ecological importance; they are one of the world’s most widely distributed plant families, and cover 31–43% of the surface of the earth (Gibson 2009), forming the foundation of major biomes such as grasslands, savannas, and steppes. They are by extension vital to many ecosystems and play a significant role in ecological stability. The evolutionary success of Poaceae is rooted in key adaptations, including the multiple independent occurrences of C4 photosynthesis (Huang et al. 2022), efficient water use strategies (Yamauchi et al. 2020), and root anatomical adaptations (Yamauchi et al. 2020; Schneider et al. 2021) that enhance soil resource acquisition and resilience. These traits enable grasses to thrive under a wide range of environmental conditions, including marginal and degraded soils (Patra et al. 2021). Here, we explore a plant composite tissue, the root cortex, as a major driver of Poaceae adaptation and resilience. We discuss the major cortical cell tissues (i.e. one or more cell types in the cortex that function together as a unit), the genotypic as well as spatiotemporal diversity in their presence, their function to the plant, and their importance for stress resilience. The focus of this review of root cortical tissues is primarily their physiological function, with special reference to their function in improving root activity under stress or limiting conditions, rather than their development, genetic regulation, and mechanisms of formation, though these too will be discussed where pertinent to function. As most of the structural and functional characterization of cortical tissues has been performed in annual grain crops in the Poaceae family, the review will focus on these species, however concepts discussed may be generally extrapolated across the family (Esau 1965; Raechal and Curtis 1990; Mallett 2002; Kellogg 2015; Cox 2017). A glossary of relevant terms and their definitions in context relevant to this review can be found in Table 1.

**Table 1:** Glossary of terms describing the composition of the poaceae root system and phenomena occurring within the root

Root Types and Categories	
Axial root	A root directly connected at the base to the seed or non-root plant body
Lateral root	A root branching from other root tissue
Adventitious root	An axial root emerging from post-embryonic tissue, in the context discussed here, stem nodes
Crown root	Adventitious roots emerging underground
Brace root	Adventitious roots emerging aboveground
Seminal root	Embryonic roots emerging from the seed
Cortical Tissues	
Cortical Tissue	One or more cell types functioning together as a unit in the cortex
Endodermis	The innermost layer(s) of the cortex which contains a Casparian strip and suberin lamellae
Parenchyma	The cortical cell type that has not undergone any further cell wall development, specialised cell divisions, senescence, or apoptosis. Characterized by a relatively thin primary cell wall made of cellulose, pectin, and hemicellulose and large intercellular spaces between them
Aerenchyma	Tissue containing enlarged gas spaces, exceeding in size those commonly found as intracellular spaces
Sclerenchyma	A specialized cortical cell, in single or multiple layers, that contains uniformly deposited lignin in cell walls
Multiseriate Cortical Sclerenchyma	Multiple layers of small cells with thick, lignin-encrusted cell walls located in the outer cortex of nodal roots (i.e. a sub-type of sclerenchyma, defined by its position in the cortex and small cells)
Phi thickenings	A lignified anticlinal band, with a partial or full radial shape that can be elaborated into reticulated structures. Note: in Poaceae this has only been documented in epidermal tissue
Hypodermis	The sub-epidermal cell layer(s) of unspecialized cortical parenchyma
Exodermis	The outermost layer of the cortex that can contain lignified and/or suberized cell wall thickenings
Cortical Regions	
Inner Cortex	The inner layer(s) of the cortex containing the endodermis
Mesodermis	The middle layer(s) of the cortex, which can be a multi-structured tissue layer
Outer Cortex	The outer layer(s) of the cortex containing the hypodermis. The hypodermis may become specialized through the deposition of lignin and/or suberin in cell walls to form an exodermis or lignin to sclerenchyma
Phenomena (in the cortex, and more broadly, the plant)	
Senescence	The process of gradual deterioration in cells, tissues, or organisms over time, leading to functional decline and, ultimately, death often driven by programmed cell death
Root Cortical Senescence	The senescence of most or all cortical parenchyma cells, common in temperate cereals
Ephemerality	The tendency for something to be short lived; in the instance of root traits, especially when viewed relative to neighbouring
Plasticity	Expression of an altered developmental pathway in response to an environmental cue
Metabolic/Tissue cost	Metabolic/tissue cost refers to the carbon and nutrient resources required to build and maintain a certain amount of root tissue

### The Poaceae root system

**Fig. 1:**
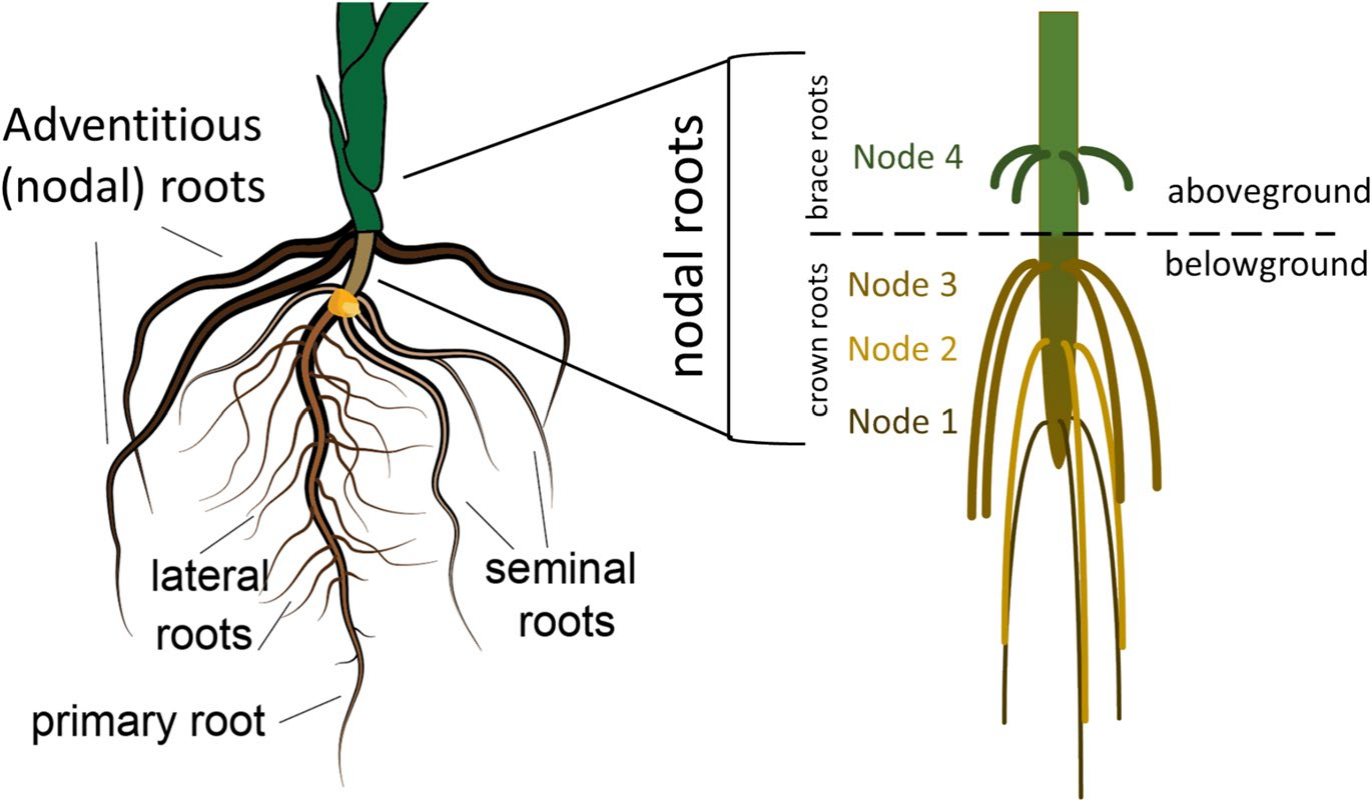
Overview of Poaceae root architecture. The primary and seminal roots emerge from the seed and comprise the embryonic root system. Later in growth, nodal roots emerge from stem nodes belowground (crown roots) and aboveground (brace roots) to make up the post-embryonic root system. Lateral roots (root-borne) can emerge from all root types. This image is a depiction of maize, but this root architecture is broadly similar across all Poaceae. The number of stem nodes developing adventitious roots above- and below-ground varies. Image modified from Kawa and Brady (2022)

Characterized by a fibrous root architecture, the root system is highly conserved across the Poaceae in its development, architecture, and anatomy. The root system begins with the emergence of the primary root and, in many cases, is shortly followed by seminal roots (together forming the embryonic root system), then is often later on expanded through post-embryonic or adventitious (derived from e.g. stem, rhizome, stolon; also referred to as nodal roots) roots. Once the shoot has emerged from the coleoptile, stem nodes produce adventitious roots sequentially, with the oldest and basal stem nodes giving rise to roots earliest and then progressively up the stem. Many grasses produce shoot branches (tillers, rhizomes, and stolons), which in turn can also produce adventitious roots. When these adventitious roots are produced from nodes below the soil line, they are referred to as crown roots, and if above, they are called brace roots (sometimes also referred to as prop or stilt roots) (Sparks 2023). Axial roots (those emerging directly from the seed or non-root plant body, both embryonic or post-embryonic), can branch to form lateral roots. Depending on the species, lateral roots can branch multiple times, producing several orders of succeeding lateral roots (Fig. 1).

**Fig. 2:**
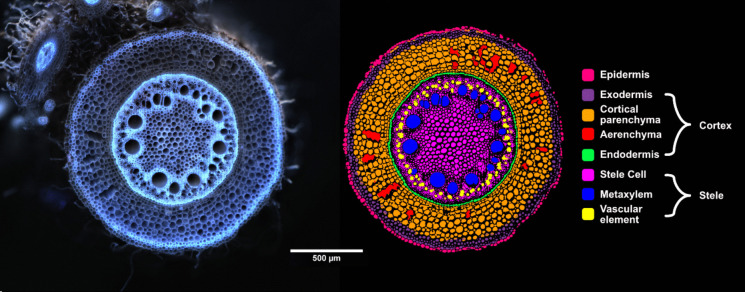
Pearl millet nodal root transverse section and annotated cell type mask. Root anatomy image captured using laser ablation tomography, and annotated using root anatomical analysis software presented in Affortit et al. (2024), scale bar = 500 µm

Axial root anatomy in the grasses varies between and within species, though it does follow certain general patterns that are consistently expressed across the family (Evert 2006). In the transverse dimension, we generally consider three zones of tissues: the stele (vascular cylinder), the cortex (ground tissue), and the epidermis. Often the primary and seminal roots possess a single, or very few, central metaxylem vessel(s) surrounded by a ring of phloem and xylem vessels. Often subsequently formed adventitious roots show an increased stele size with multiple files of metaxylem vessels, arranged in a polyarch ring, surrounded by an outer ring of phloem and xylem vessels, but can also contain just a single meta-xylem, which is occasionally observed in both cultivated and wild grasses across a range of environments (Rogers and Benfey 2015; Passot et al. 2016; Hochholdinger et al. 2018; Liu et al. 2020; Yamauchi et al. 2020; Fusi et al. 2024) (Fig. 2). The anatomy of lateral roots is consistent with axial roots in their composition of a stele, cortex, and epidermis, in the same layered sequence, though the vascular anatomy and overall size can vary widely even within lateral roots of similar ages branched from the same parent root (Cox 2017). Although root anatomy is organizationally highly conserved in the Poaceae, the combinations of the presence, size, and distribution of different tissues and cell types and the timings of their development and fate, reveals significant diversity upon investigation. In all of these described root types, the cortex (as will be discussed) is highly dynamic and can even be ephemeral in nature.

## The cortex

The cortex of the plant root is the composite layer of tissue(s) that sits between the central vascular tissue and the epidermis, serving as a critical interface between the soil environment and the vascular tissues. Cortical cell arrangement of axial roots varies between species, presenting as ordered concentric rings and radial columns or as less regular structures, with cells spanning radii and layers. In the Poaceae, the cortex typically consists of multiple layers of parenchyma cells arranged concentrically around the vascular cylinder. Here the term ‘cortical parenchyma’ describes the cortical cell ‘type’ that has not undergone any further cell wall development, specialized cell divisions, senescence, or apoptosis. Parenchyma cells are often characterized by their relatively thin primary cell wall made of cellulose, pectin, and hemicellulose and large intercellular spaces between them (Krishnamurthy et al. 2015).

**Fig. 3:**
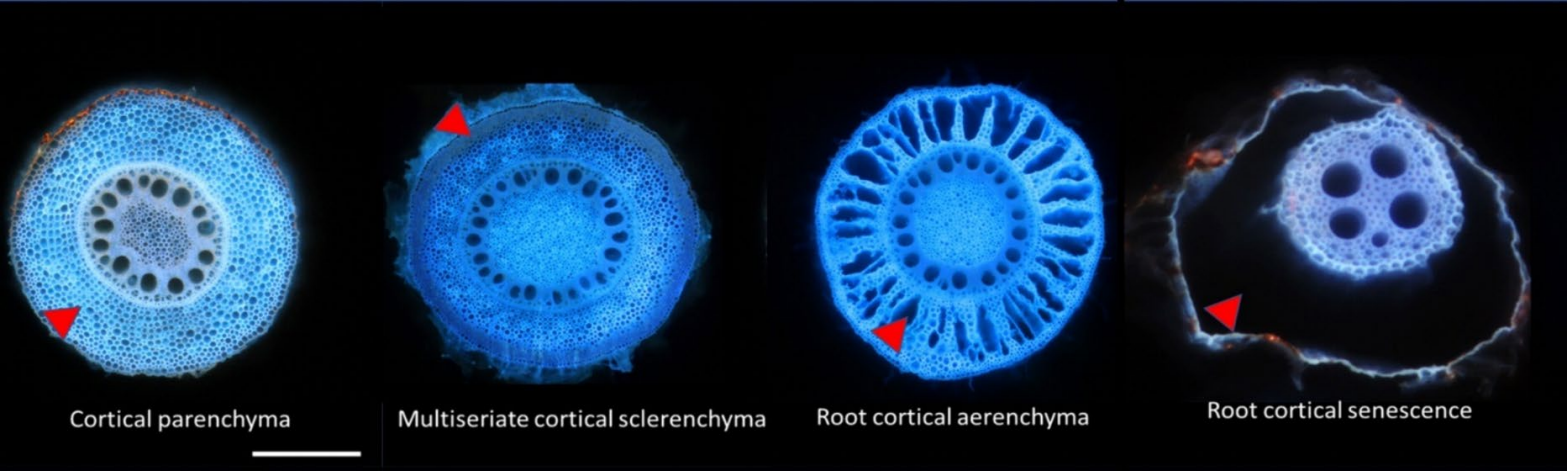
Different tissues can be developed in the Poaceae axial root cortex through divergent trajectories of cortical parenchyma (maize) marked in red arrows including multiseriate cortical sclerenchyma (maize), root cortical aerenchyma (maize), and root cortical senescence (wheat). Scale bar = 0.5 mm

The cell identity of ground tissue (e.g., cortical parenchyma) has been well studied in Arabidopsis, where one initial cell forms all tissues of the ground tissue (Dolan et al. 1993; Campilho et al. 2006). The *SHORT-ROOT* (*SHR*) and *SCARECROW* (*SCR*) genes, both members of the plant-specific GRAS transcription factor family, play important roles in the division forming the cortical layer and the cell identity of the resulting cells. For example, *scr* and *shr* mutants in Arabidopsis develop only a single ground tissue layer (Scheres et al. 1995; Di Laurenzio et al. 1996). In the *shr- 1* mutant, this monolayer adopts a cortex identity, whereas in *scr- 1*, the single layer exhibits a mixed endodermal and cortical fate (Benfey et al. 1993; Di Laurenzio et al. 1996; Heo et al. 2011). While seedling roots of Arabidopsis have only two ground tissue layers (one layer of cortical parenchyma and one layer of endodermis), the roots of grasses typically have multiple cortical cells layers, ranging from 6 to 20 layers in maize, 5 to 10 layers in rice, 4 to 6 in *Brachypodium* (Fusi et al. 2024) and 4 to 5 layers in green foxtail (*Setaria viridis)* (Clark and Harris 1981; Ortiz-Ramírez et al. 2021). In rice, the genesis of the multilayered cortex is the result of periclinal divisions of the cortex-endodermal-epidermal-initial cell line (CEEID) (Pauluzzi et al. 2012), however cortical cell identity across Poaceae species is not well understood. Recently, several studies have tackled the genetic underpinnings of the multilayered cortex in Poaceae, including describing the movement of *SHR* from the stele further out into the cortex and the roles SCR and SHR in determining the number of cortical cell file layers and cortical cell identity (Pauluzzi et al. 2012; Ortiz-Ramírez et al. 2021),

Soon after root development, the root cortex in Poaceae is predominantly composed of parenchyma cells (Esau 1965; Mallett 2002; Kellogg 2015). However, as the axial root matures and encounters diverse environmental conditions, the cortex undergoes significant structural and functional modifications (described below). These changes are mediated through the deposition of key structural compounds like lignin and suberin within the cell walls, and through programmed cell death in spatial and temporal patterns (Fig. 3). Through these modifications, which are often observed to a greater or lesser extent in different species, the otherwise largely homogenous cortex of the Poaceae can present with significant diversity in form and function. Poaceae do not possess the capacity for secondary growth (i.e., radial expansion) within their roots, so do not tend to ‘shed’ their cortex as early or in the same way as other plant families (e.g., Fabaceae). However, individual parenchyma cells and cell file layers may be ephemeral (i.e. short lived relative to other root tissues) in Poaceae due to divergent developmental trajectories. Groups of cells that acquire cell wall alterations or undergo programmed cell death become functionally delineated as a different tissue type or structures.

Cortical tissues and structures and their respective functions vary between species, root types, and root zones, depending on environmental conditions (Ogorek et al. 2023). The size and number of cortical layers, along with stele size, determine overall root diameter. Root diameter is particularly significant as it influences the surface area available for nutrient uptake, which is further expanded by the epidermis, root hairs, and symbiotic associations. Because the stele consists mainly of lignified vasculature with minimal metabolic activity (Schneider et al. 2017b), and since the cortex often occupies a larger cross-sectional area relative to the stele, cortical metabolic demands can be substantial (Sidhu et al. 2024). These demands, primarily for tissue construction and maintenance, can consume over 50% of the daily net carbon fixed by the shoot (Nielsen et al. 1998; Fan et al. 2003) In nutrient-poor environments, plants allocate an even greater proportion of their photosynthates to root systems to enhance nutrient acquisition (van der Werf et al. 1988; Lambers et al. 1996; Nielsen et al. 1998, 2001). However, this resource allocation comes at the cost of reduced photosynthetic tissue growth, ultimately affecting yield. Since cortical tissues significantly influence root maintenance costs, the development of tissues with reduced carbon and nutrient requirements can help reduce the plant’s overall metabolic burden (Lynch et al. 2021).

Beyond determining the carbon and nutrient costs of the root, cortical tissues also regulate the transport of water, nutrients, and gases through the root system and interact with soil biota. For example, the arrangement of parenchyma cells, aerenchyma, and incidence of cortical senescence in Poaceae influences radial resource movement by modifying symplastic and apoplastic pathways in the cortex (Fan et al. 2003; Hu et al. 2014; Schneider et al. 2017b). Additionally, apoplastic barriers such as a lignified Casparian strip and suberin lamellae in the endodermis and exodermis, and lignified polar cap or uniformly lignified cells in the exodermis restrict passive water and solute movement, necessitating active transport mechanisms (Schreiber and Franke 2011; Manzano et al. 2025; Cantó-Pastor et al. 2025). Moreover, variation in cortical area in maize affects arbuscular mycorrhizal fungi colonization, altering phosphorus uptake (Galindo-Castañeda et al. 2022).

Here we review the structure and function of cortical tissues primarily within the context of the axial root as this has been the subject of the greatest body of characterization work. We describe tissues beginning with the innermost layer(s) of the cortex, the endodermis, to the outermost layer(s), the hypodermis, and the potential tissues in between, formed by divergent developmental trajectories of cortical parenchyma cells.

## Inner cortex

### Endodermis

The endodermis is typically a single cell layer in Poaceae, defined by its position as the innermost layer of cortical cells, so is inherently highly conserved in its presence, form, and function, across all the Poaceae. The current understanding of the differentiation and function and the molecular network controlling these aspects of the endodermis, particularly regarding nutrient transport, has been thoroughly reviewed (Enstone et al. 2002; Robbins et al. 2014; Barberon et al. 2016; Barberon 2017; Andersen and Drapek 2024). Here, we mainly focus on the endodermis through the lens of the Poaceae and its importance when considering the dynamics of other cell types and tissues in the root cortex.

**Fig. 4:**
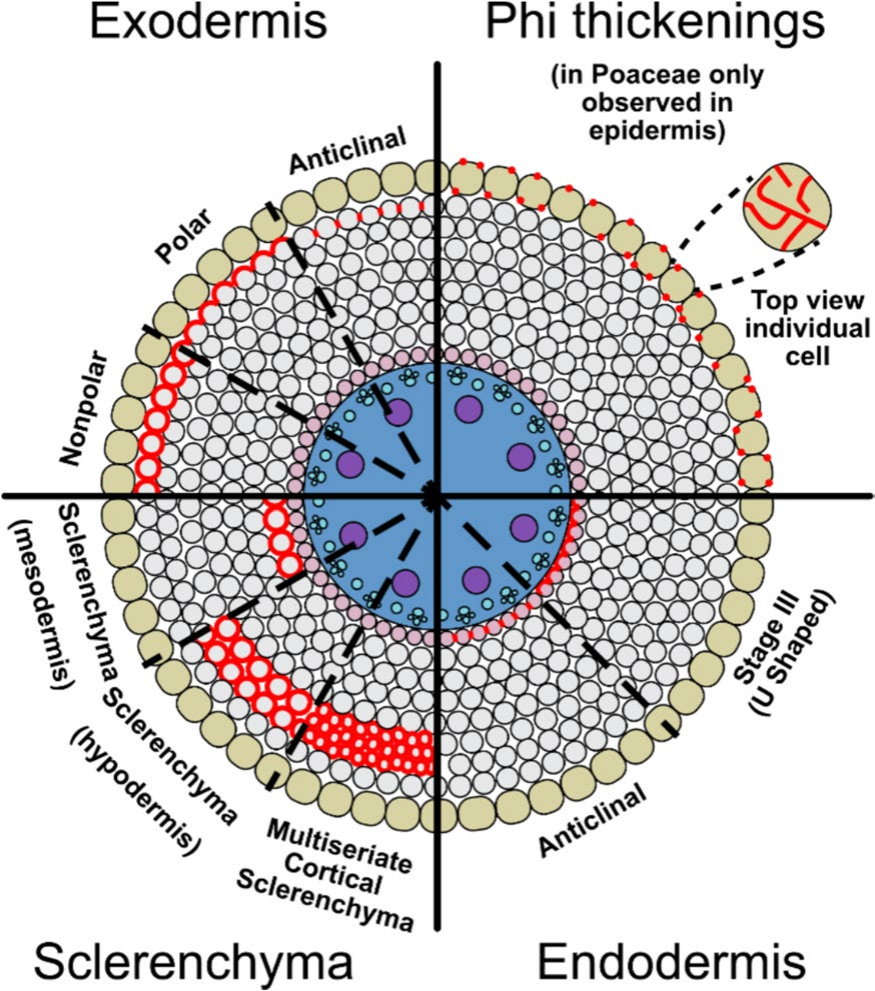
Overview of cell wall thickenings in Poaceae depicted on a transverse cross-section. Lignin is shown in red. The three patterns of lignin deposition in the exodermis of Poaceae include nonpolar (entire exodermal cell is surrounded by the barrier), polar cap (barrier is polarized to the periclinal cell wall), and anticlinal (barrier deposited in anticlinal walls). Phi thickenings are in the radial and transverse walls. In Poaceae phi thickenings have not been observed in the cortex, but in the epidermis. In the endodermis the Casparian strip is deposited in anticlinal walls and can progress into stage III (U-shaped) thickening. Sclerenchyma cells have a uniform thickening of lignin that can be deposited in the mesodermis (adjacent to the endodermis) or in the hypodermis. Multiseriate cortical sclerenchyma, is a type of sclerenchyma in the hypodermis, but is characterized by a small cell size. Epidermal tissues are marked in green, cortical tissues in grey and pink (endodermis), and vascular tissues in blue. Adapted from Cantó-Pastor et al. 2025 (Clark and Harris 1981; Bennett 1982; Grymaszewska and Golinowski 1987; Kawa and Brady 2022)

Unlike the exodermis, which is absent in some species of Poaceae (described below), the endodermis is a universal feature in Poaceae and serves as the main apoplastic barrier. Two key structural components characterize the mature endodermis: Casparian strip and suberin lamellae. Endodermal differentiation can be categorized into three stages. In stage I, the Casparian strip, a ring-like, apoplastic barrier layer made of lignin, may seal the intercellular spaces between the endodermis cells, meaning water and transported solutes must enter the plant cell cytoplasm to move across the endodermis layer. Casparian strip formation is synchronized across the entire layer, creating a network that isolates the stele’s apoplast from outer tissues. After Casparian strip formation, the endodermis undergoes a second differentiation phase (stage II), where suberin, a hydrophobic secondary cell wall polymer, is deposited between the plasma membrane and primary cell wall, completely covering the surface of endodermal cells. Depending on the maturation, a small number of cells in the endodermis, named passage cells, remain non-suberized, forming a water transport route in fully suberized endodermis (Andersen et al. 2018). In certain species, such as sorghum, maize, and sugarcane, endodermal cells can also reach developmental stage III, characterized by U-shaped thick secondary cell walls composed of lignin and carbohydrates (Zeier and Schreiber 1998; Enstone et al. 2002), and sugarcane also has central projections in this U-thickening pattern (Edson-Chaves and Graciano-Ribeiro 2018) (Fig. 4). The regulation of the different stages of endodermal differentiation has been extensively investigated, revealing a suite of genes governing the molecular processes critical to each stage (Ortiz-Ramírez et al. 2021; Yang et al. 2022; Shaar-Moshe and Brady 2023). In the formation of the endodermal cell file (and increased number of cortical layers), *SHR* and *SCR* genes are critical in rice, Setaria, and maize (Ortiz-Ramírez et al. 2021) and presumably most members of the Poaceae family. Genes involved in Casparian strip and suberin lamellae deposition are also well understood in a range of species including sorghum, maize, and rice (Wei et al. 2020; Wang et al. 2022; Yang et al. 2022).

The specific roles of endodermal Casparian strip and suberin lamellae in controlling radial water and solute movement has been widely debated (Peterson et al. 1993; Steudle et al. 1993; Frensch et al. 1996; Geldner 2013; Nawrath et al. 2013; Doblas et al. 2017) and references within). Additionally, new findings in Arabidopsis suggest that disruptions in Casparian strip formation can trigger signalling pathways that influence aquaporin activity and hydraulic responses (Wang et al. 2019). While the cell wall composition of the endodermis, including lignin and suberin, can change over time, its anatomy is relatively consistent, showing less variation than other cortical cell types and tissues. However, since Casparian strip and suberin lamellae are crucial in regulating plant water relations and nutrient uptake, relatively minor alterations in these structures, including the amount of biopolymer deposition and timing of differentiation, affect shoot ion composition, stress tolerance, and shoot development. For example, mutants in *ZmSTL1* (*Salt-Tolerant Locus 1*) in maize have been shown to reduce lignin deposition in the Casparian strip in the endodermis, leading to a defective barrier and increasing the apoplastic transport of Na + across the endodermis (Wang et al. 2022). A greater expression of suberin biosynthesis genes triggered by low nitrate supply resulted in enhanced endodermal suberization preventing radial transport of Cd in maize roots (Chen et al. 2023). In barley, osmotic stress resulted in the upregulation of genes in the suberin biosynthesis pathway and resulted in increased suberin in the endodermis and a reduction in hydraulic conductivity (Kreszies et al. 2019). In addition, saline conditions (Karahara et al. 2004) and water-stressed conditions (Líška et al. 2016) in maize can trigger endodermal differentiation closer to the root tip or when exposed locally can trigger asymmetrical differentiation of the endodermis (Karahara et al. 2004).

In addition to the deposition of the Casparian strip and suberin lamellae, some Poaceae, including sorghum, sugarcane, wheat, and rice (Lux et al. 2020) also commonly have silica impregnated in cell walls of the endodermis and exodermis (Soukup et al. 2017). The presence of silica deposits in roots can improve mechanical properties of tissues and is linked to stress tolerance (Ma 2004; Hodson et al. 2005; Cooke and Leishman 2011; Liang et al. 2015). Silicon deposits in the cortex may enhance the mechanical strength and viscoelasticity of cell walls, protect against pathogens, mitigate stress from drought and metal(loid)s, facilitate metal coprecipitation, and regulate nutrient uptake (Coskun et al. 2019; Lukacova et al. 2023). However, the function of silica in the roots has been widely debated (Coskun et al. 2019), especially under non-stress conditions, with some studies indicating positive effects on growth and yield, while others report conflicting results (Coskun et al. 2019). For example, silica aggregation in sorghum is common and takes place in non-lignified positions in endodermal cell walls, progressively accumulating into silicic acid and initiating arabinoxylan–ferulic acid complexes, reducing water permeability, preventing water loss, and improving drought resistance (Lux et al. 2020). Other studies demonstrated that in sorghum, silicification of the endodermis did not influence radial water loss, however it reduced root growth inhibition caused by desiccation (Soukup et al. 2017). In rice, silicon weakens and delays the formation of the barrier to radial O_2_ loss and leads to an increase in Na⁺ and Cl⁻ concentrations in the xylem sap (Tong et al. 2024). Silicon supplementation, whether under control conditions or osmotic stress, does not increase suberization in barley roots (Kreszies et al. 2020b). Overall, while silicon deposition in roots is associated with various structural and physiological benefits, its precise role, particularly under non-stress conditions, remains unclear, warranting further investigation to fully understand its impact on tissue function and plant resilience.

The environmental dependency of suberin differentiation in Poaceae species is also demonstrated with respect to biotic interactions (Kawa and Brady 2022). The soil microbiome has been shown to induce suberization of endodermis in sorghum crown roots (Kawa et al. 2024) and individual bacterial isolates were identified to elicit increased suberin levels in sorghum and rice roots (Kawa et al. 2024; Alghamdy et al. 2024). These observations contrast with findings in Arabidopsis, where several soil-borne bacteria weakened endodermis apoplastic barriers, by decreasing suberin content and only very few bacterial strains increased root suberization (Salas-González et al. 2021). Altogether it appears that soil microbiome shapes endodermal suberized barriers in monocot and dicot species in an opposite manner. However, the number of isolates tested thus far in Poaceae species is much lower than those tested in Arabidopsis, raising the need for more systematic characterization of soil-borne bacteria on root suberization.

Endodermis cell wall modifications are not only under the influence of commensal and beneficial bacteria but can also act as a physical barrier to pathogens. The endodermis of sorghum, maize, and rice increase in biosynthesis of lignin, suberin, and unknown phenolic compounds, as well as silica deposition, preventing the major cereal pest, *Striga hermonthica*, from reaching its host vasculature (Kawa and Brady 2022; Kawa et al. 2024). Consequently, varieties with an “enforced” endodermis show increased resistance to Striga (MAITI et al. 1984; Gurney et al. 2006; Yoshida and Shirasu 2009; Cissoko et al. 2011; Mbuvi et al. 2017; Mutinda et al. 2018; Mutuku et al. 2019). Altogether, endodermis differentiation is not only shaped by the biotic agents, but its differentiation status serves as a protective layer from pests.

While there is a significant understanding of the development, regulation, and function of the endodermis in Arabidopsis, and it has been shown that several of these identified regulatory pathways are conserved in other species, overall, far less is known about this tissue in the grasses. Further research is also needed to understand the individual contributions of the Casparian strip and suberin lamellae to the function of the endodermis as an apoplastic barrier, which is challenging due to mutually compensatory mechanisms (Shukla et al. 2021; Grünhofer et al. 2024).

## Mesodermis

The mesodermis refers to the middle layer(s) of the cortex, which can be a multi-structured tissue layer, therefore it will be discussed more through the review of its constituent cell and tissue types. The mesodermis is bounded by the endodermis, and the exodermis or sclerenchyma where present, or the hypodermis if not. The mesodermis is structurally versatile and can differentiate into various tissues based on environmental and developmental cues. These include aerenchyma, which aid in gas exchange by forming air spaces through schizogenous or lysigenous processes or parenchyma cells can senesce to form root cortical senescence reducing the metabolic cost of soil exploration. The ability of the mesodermis to develop different tissues contributes to its functional flexibility, making it an essential component of root structure in Poaceae.

### Cortical parenchyma

In Poaceae roots, individual parenchyma cells and cell layers may maintain their identity as parenchyma cells in their developmental trajectory, retaining their functional roles and relatively simple structure. These cells facilitate the movement of water and nutrients from the outer tissue layers of the root to the innermost cortex (endodermis), and through to the stele (Lux et al. 2004a, b). Cells that maintain their identity as cortical parenchyma have a high capacity for radial water transport when considered in the composite transport model. Having no cell wall modifications or aerechymatous lacunae means water can travel through the cortex apoplastically, symplastically, and intercellularly without severe modifications in the path lengths of transport (Kim et al. 2018; Couvreur et al. 2018). This persistence of parenchyma can be particularly advantageous in some environments to mediate and regulate radial movement of water and solutes, serve as a storage location for reserve materials, play a role in pathogen resistance and symbiotic interactions, and for the synthesis of several secondary metabolites. For example, calcium aids in structural integrity in the cell wall, or is stored in the vacuole. Bivalent or multivalent ions such as iron, manganese, and zinc (Robertson 2013; Bhatla and Lal 2023) can be bound to the cell walls or bound to chelators in the vacuole to aid in enzymatic reactions and redox balance (Wink 1993). Additionally, persistent cortical parenchyma may develop select modifications and deposits in a similar capacity to the endodermis. Cortical parenchyma in Poaceae have the capacity to deposit silica bodies or phytoliths, contributing to structural support and defence against herbivory (Nawaz et al. 2019).

In addition, cortical parenchyma has key contributions to the biochemical synthesis of specialized metabolites, including phenolics (lignin precursors, flavonoids), alkaloids, and hormones. Lignin precursors, primarily monolignols, are synthesized via the phenylpropanoid pathway in the cytoplasm of cortical and vascular parenchyma cells, before being transported to the apoplast for polymerization into lignin (Barrière et al. 2007). Flavonoids, including flavones and isoflavonoids, are also synthesized in the cortical parenchyma and epidermal cells, playing crucial roles in root exudation, rhizosphere signalling, and stress responses (Maier et al. 1995). Plant hormones, including auxins and abscisic acid, are synthesized in specific root tissues: auxins primarily in pericycle and cortical parenchyma cells, and abscisic acid in parenchyma and endodermal cells in response to stress (Yue et al. 2021; Wakeman and Bennett 2023). Additionally, strigolactones, important for mycorrhizal symbiosis and the regulation of shoot growth, are predominantly synthesized in root cortical parenchyma and the pericycle. All these specialized metabolites contribute to root development, environmental adaptation, and interactions with the rhizosphere, highlighting the role of cortical parenchyma as a metabolic hub important for plant function and stress resilience (Guan et al. 2012; Ravazzolo 2019).

Nevertheless, even prior to the onset of differentiation programs of cortical parenchyma, different subdomains, demarking an inner and outer cortical subdomain beyond the endodermis, have been identified in rice through immunoprofiling (Henry et al. 2016) and transcriptional profiles of different cortical layers have been shown to be unique (Ortiz-Ramírez et al. 2021). This provides an opportunity to gain further insight into the subtle foundations that may be in place preceding further differentiation of cortical parenchyma, and the mechanisms determining the specific spatial patterns of these processes. Many aspects of cortical parenchyma development have been shown to be under genetic control. Large genomic and phenotypic screens of maize roots revealed association between the number of cortical cell file layers, as well as cortical cell size, and total cortical area, and a broad suite of candidate single nucleotide polymorphisms (Schneider et al. 2020a, b). Furthermore, this screen revealed different regulation of these cortical parenchyma traits under stress and controlled conditions, as well as for the plasticity of these traits. Though integrated as part of a trait measuring whole cortex area, unique quantitate trait loci were identified in barley associated with the water deficit response of root cortical area of adventitious roots of the main stem and tiller stems (Oyiga et al. 2020).

However, in many cases parenchyma cells can have divergent developmental trajectories to form different tissues through spatial and temporal patterns of lignin and/or suberin deposition in cell walls or programmed cell death. Parenchyma cells can develop combinations of these tissues simultaneously and successively in the root cortex. Since cortical parenchyma have important functions for the root organ and plant, the transition of parenchyma into different cortical tissues changes cortical function and alters plant stress tolerance. The impact of cortical tissues on stress tolerance varies depending on the specific (or combinations of) tissue and the nature of the environmental stress.

### Aerenchyma

Parenchyma cells may develop tissue containing enlarged gas spaces, exceeding in size those commonly found as intracellular spaces, to form root cortical aerenchyma as part of their development trajectory (Kawai et al. 1998). Root cortical aerenchyma can be induced or formed constitutively in grasses to facilitate gas exchange in waterlogged or oxygen-deficient soils (Jackson et al. 1985; Thomson et al. 1990; Manik et al. 2022). Beyond hypoxia, root cortical aerenchyma can be triggered by various abiotic stresses, including drought (Zhu et al. 2010; Chimungu et al. 2015), heat (Hu et al. 2014), and limited availability of nutrients such as nitrogen (Saengwilai et al. 2014), phosphorus (Fan et al. 2003; Galindo-Castañeda et al. 2018), and sulphur (Bouranis et al. 2006). Moreover, biotic interactions can also promote aerenchyma formation, as seen in sorghum nodal roots, where aerenchyma was formed in plants grown in field-collected soil, but was absent when the same soil was sterilized (Kawa et al. 2024). Aerenchyma can be schizogenous (formed through cell separation), lysigenous (formed through cell death), or expansigenous (formed through cell division and expansion) in origin (Seago et al. 2005). Most documented aerenchyma in Poaceae is lysigenous (Evans 2004), but schizogenous development has been noted in some species including young rice roots (Qu et al. 2016).

Schizogenous aerenchyma forms through the separation of cells within the cortical tissue. This process involves the loosening of cell walls and the dissolution of middle lamellae, creating intercellular spaces without the death of the surrounding cells. In contrast, lysigenous aerenchyma arises from the programmed cell death of groups of cortical cells, resulting in large air-filled lacunae (varying in size and shape) separated by strands of living cells (resembling a"spoked wheel"arrangement) to help maintain root structural integrity and ensuring radial nutrient and water transport (Drew and Fourcy 1986). Lysigenous aerenchyma are common in both temperate and tropical cereal crops, including but not limited to, wheat, barley, rice, maize, and sorghum (Yamauchi et al 2013). In Poaceae, lysigenous aerenchyma formation is regulated by hormonal signalling, particularly involving ethylene, reactive oxygen species, and calcium-dependent pathways. In rice, which shows constitutive aerenchyma formation, aerenchyma has been shown to be regulated by auxin and auxin response factor signalling. This same signalling pathway was shown to also effect lateral root formation, but lateral root formation preceded aerenchyma, suggesting differential regulation of lateral roots and aerenchyma in response to the auxin signal (Yamauchi et al. 2019). A maize relative, *Zea nicaraguensis* was also shown to utilize auxin signalling in patterning aerenchyma with regard to lateral root emergence and gravistimulus responses (Ning et al. 2023). In maize, the bHLH121 transcription factor has been shown to be an important regulator of root cortical aerenchyma formation (Schneider et al. 2023). These signals trigger programmed cell death, enabling the plant to adapt to stress conditions by creating low-resistance pathways for gas diffusion while reducing metabolic demands of root tissues (Evans 2004). As aerenchyma can be constitutive, or induced in response to a range of abiotic factors (Pedersen et al. 2021), and there are diverse hormonal and transcriptional regulators of their formation. For a thorough overview of the current status of our understanding of the mechanisms underpinning aerenchyma formation, see Yamauchi and Nakazono (2022a, b). In most scenarios however, key aspects of this mechanism are still unresolved. The formation of aerenchyma can be highly spatially and temporally heterogeneous, affected by many different signalling pathways with shared responses to a myriad abiotic factors.

The functional benefits of aerenchyma are significant for plants growing in stress-prone environments. In a variety of species, constitutive aerenchyma formation are commonly seen and are often described as part of the plant root systems acclimation and adaptation to the environment. This is especially well noted in rice (Clark and Harris 1981; Yamauchi and Nakazono 2022a, b), though is also observed in *Tripsacum dactyloides* (a forage grass) (Ray et al. 1998; Swift et al. 2025), and in the maize relative, *Zea nicaraguensis* (Mano and Omori 2013). For example, lysigenous root cortical aerenchyma formation under nutrient and water stress is considered adaptive, as it reduces the metabolic costs of soil exploration by converting living cortical parenchyma into gas spaces (Fan et al. 2007). In maize, root cortical aerenchyma formation under nutrient stress was linked to lower root respiration, increased root growth, enhanced nitrogen and phosphorus capture, improved shoot nutrient status, and greater photosynthesis, growth, and yield (Saengwilai et al. 2014; Galindo-Castañeda et al. 2018). These benefits were driven by reduced root respiration and nutrient reallocation from cortical tissue into further root and shoot growth (Postma and Lynch 2011). Similar advantages were observed under drought stress, where root cortical aerenchyma development in maize was associated with reduced root respiration, deeper rooting, better shoot water status, improved leaf photosynthesis, and increased growth and yield (Zhu et al. 2010). A recent study in maize also linked increased root cortical aerenchyma formation, in combination with other cortical and vasculature traits, to drought adaptation (Klein et al. 2020). Moreover, presence of aerenchyma has been associated with lower infection levels with *Fusarium verticilloides* in maize and *Striga hermonthica* in sorghum (Kawa et al. 2024). This suggests that aerenchyma can also protect Poaceae from biotic stress by preventing pathogens from progressing radially through the root. Aerenchyma formation comes at the cost of a reduction in the amount of and overall function of cortical parenchyma. Presence of aerenchyma has been shown to restrict radial water transport and decrease overall water and nutrient uptake (Gao et al. 2020; Basu et al. 2021; Yang et al. 2012; Hu et al. 2014). As aerenchyma formation is a common response to water deficit stress, this may serve not only to reduce root metabolic cost, but to conserve available soil water resources.

### Root cortical senescence

Unlike aerenchyma, which forms discrete lacunae, root cortical senescence results in senescence of most or all cortical parenchyma cells in several species including triticale (*Triticosecale*) (Liljeroth and Bryngelsson 2001, 2002), barley (Henry and Deacon 1981; Liljeroth and Bryngelsson 2002), rye, oats (Liljeroth 1995), Kentucky bluegrass (*Poa pratensis*) (Smiley 1986), perennial ryegrass (Jupp and Newman 1987), blue grama grass (*Bouteloua gracilis*) (Beckel 1956), and wheat (Henry and Deacon 1981; Lascaris and Deacon 1991; Liljeroth and Bryngelsson 2002). Root cortical senescence resembles lysigenous root cortical aerenchyma in its formation via programmed cell death but differs in its more limited taxonomic distribution, being observed primarily in temperate Poaceae species, while aerenchyma formation is a broader adaptation across most plant taxa.

Root cortical senescence follows a spatiotemporal pattern, initiating in outer cortical cell files near the zone of anucleate root hairs and spreading inward and basipetally, resulting in anucleate cortical cells. Over time, nearly all cortical cells within axial roots, from just behind the apex to near the root-shoot junction, become anucleate and senesce, typically not including the endodermis, or often the cortical cell layer adjacent to the endodermis (Henry and Deacon 1981; Schneider et al. 2017b). Root cortical senescence culminates in a non-continuous epidermis, which also eventually senesces. The occurrence root cortical senescence is associated with increased aliphatic suberin in the endodermis (Schneider et al. 2017b). Root cortical senescence progression varies by species, environmental, and genetic factors (Liljeroth and Bryngelsson 2002; Schneider et al. 2017b), with wheat experiencing the fastest progression, followed by triticale, barley, rye, and oats (Henry and Deacon 1981). In wheat grown in soil, 80–90% of cortical parenchyma cells (cross-sectionally) senesce, compared to 20–35% in barley and rye (Liljeroth 1995). Ethylene exposure and nutrient deficiency accelerate the progression of root cortical senescence (Schneider et al. 2018). However, despite identifying genetic variation, mechanisms controlling root cortical senescence are not well characterized.

Root cortical senescence has been demonstrated to enhance nutrient deficiency and drought resilience. Root cortical senescence reduces the carbon and nutrient costs of soil exploration by minimizing the metabolic demands of maintaining living parenchyma cells (Schneider et al. 2017b). This reallocation of resources supports shoot growth and new root development, enhancing soil exploration. Under nutrient-limited conditions, plants with root cortical senescence had longer roots and lower respiration per unit root length (Schneider et al. 2017a). Moreover, nutrient remobilization during root cortical senescence is particularly advantageous under nutrient stress. Although the amount of carbon and nutrients released by senescing cells might be small, over time they may have a significant effect, as an improved plant nutrient status supports greater growth rates and, thus, greater soil exploration in an autocatalytic manner. Reallocating phosphorus, nitrogen, and potassium from senescing cortical tissue increased shoot growth by 33%, 100%, and 27%, respectively, under suboptimal nutrient availability (Schneider et al. 2017a).

Root cortical senescence is also associated with reduced radial hydraulic conductivity and nutrient transport, including nitrogen and phosphorus (Schneider et al. 2017b), due to disruptions in apoplastic and cell-to-cell pathways, reduced root-soil contact, and increased endodermal suberization. However, reduced water and nutrient transport has an overall negligible effect on plant growth, as lateral roots, responsible for most water and nutrient uptake, are unaffected by root cortical senescence (Schneider et al. 2017b, 2020b). Furthermore, root cortical senescence typically occurs in older roots that have already depleted their surrounding soil of resources, minimizing its impact on overall nutrient uptake.

Root cortical senescence shares several functional similarities with lysigenous root cortical aerenchyma. Both processes are accelerated under nutrient deficiencies (Gillespie and Deacon 1988; Drew et al. 1989) involve ethylene signalling (Schneider et al. 2018), involve programmed cell death in cortical parenchyma cells, and reduce radial nutrient transport, hydraulic conductivity, and root metabolic costs (Hu et al. 2014; Saengwilai et al. 2014; Schneider et al. 2017b). However, the reductions in radial nutrient and water transport in axials roots and root respiration associated with root cortical senescence are significantly greater than those observed with root cortical aerenchyma due to proportionally greater losses of cortical parenchyma cells (Fan et al. 2007; Schneider et al. 2017b).

Despite their similarities, root cortical senescence and root cortical aerenchyma differ functionally and anatomically. Root cortical aerenchyma is a common response to hypoxic conditions, aiding gas diffusion within the root, whereas root cortical senescence does not share this function. Anatomically, the pattern of cell death is distinct between the two tissues. Root cortical aerenchyma begins in the middle cortical cell files and progresses radially, leaving strands of parenchyma cells intact to connect inner and outer cortical tissues, while root cortical senescence starts in the outer cortical cell files and progresses inward until all cortical parenchyma and epidermal tissue are senesced. Although different, root cortical aerenchyma and root cortical senescence may occur within the same root region, separated by time, both contributing to a reduction in metabolic expenditure, but with different functional costs and benefits. Further research is needed to explore the genetic and physiological differences between root cortical senescence and root cortical aerenchyma. While the development of these tissues may seem functionally redundant in many aspects, they may act in synergy when formed successively (e.g. aerenchyma transitioning into cortical senescence) for stress tolerance.

### Phi thickenings

Lignin can be deposited in parenchyma cell walls to form phi thickenings, specialized, secondary wall structures (Enstone et al. 2002, Evert 2006; Idris and Collings 2019; Aleamotuʻa et al. 2022). In many gymnosperms and some dicotyledons and monocotyledons, cortical cells may develop band-like or reticulate thickenings, called phi thickenings (in cross-sections resembling the Greek letter Φ, where thickening zone resembles the circle element, and the two wall edges, resemble the central rod), located in cortical parenchyma cell walls (Fig. 4). The development of phi thickenings is not always radially continuous and varies along the longitudinal axis of the root (Aleamotuʻa et al. 2022). Phi thickenings can be induced by a range of stresses, including drought and heavy metal toxicity (Aleamotu‘a et al. 2019). In monocots, phi thickenings are prevalent in the Orchidaceae (Porembski and Barthlott 1988; Burr and Barthlott 1991). Maize has been described as having phi thickenings in the epidermis (Degenhardt and Gimmler 2000), compared to the typical positions of type I (adjacent to endodermis), type II (hypodermis) and type III (throughout the cortex) phi-thickenings. While phi thickenings are not well documented in the Poaceae, we feel it is important and relevant to mention as they have been observed in maize and may also be present in the cortex of other Poaceae species but how frequently is unknown.

## The hypodermis and outer cortex

The outer cortex (approximately 3–5 cell files directly below the epidermis) in Poaceae roots provides essential functions such as mechanical support, regulation of water and ion transport, and protection against environmental stresses. Structurally, the outer cortical layers can vary across species but often include tissues with suberized or lignified walls, enhancing their protective and selective barrier roles. The hypodermis is comprised of sub-epidermal cell layers and is present in in various plant organs, including fruits, leaves, stems, and roots. In roots, the hypodermis represents the outermost layers of the cortex and is composed of parenchyma cells (Eshel and Beeckman 2013). While some studies have defined the hypodermis as a suberized tissue (Clarkson et al. 1987), here we define it by its position in the cortex and its persistence as unspecialized parenchyma cells. The hypodermis may become specialized through the deposition of lignin and/or suberin in cell walls to form an exodermis or lignin to form sclerenchyma.

### Exodermis

The exodermis is defined as hypodermal layer(s) with lignified cell walls or/and suberin lamellae (i.e. thickened cell walls) that may or may not act as an apoplastic barrier (Peterson and Waite 1996; Hose et al. 2001; Enstone et al. 2002) (Fig. 4). Serving as the first line of defence for water and nutrient absorption in roots, the exodermis can block the apoplast and limit the membrane area available, thereby regulating radial water, solute, and gas transport (Peterson 1989, p. 198; Enstone et al. 2002). Many Poaceae develop a uniseriate or multiseriate exodermis in a constitutive or inducible manner (i.e. forming in response to an environmental cue). Although the endodermis is present in nearly all vascular plants, certain species, including the model plant Arabidopsis, lack an exodermis and crops like wheat and barley, lack a constitutive exodermis (Perumalla et al. 1990; Kreszies et al. 2019). This absence has contributed to a comparatively limited understanding of the function of the exodermis relative to the endodermis. However, it has been demonstrated that the exodermis differs from the endodermis in terms of suberin content, microstructure, and its transport properties for water, solutes, and gases in dynamic environments, further emphasizing its critical role in root function (Ranathunge et al. 2003; Ogorek et al. 2023).

Typically, Poaceae develop a uniform exodermis consisting of a single cell type and complete plasmodesmatal connectivity between exodermal cells as in maize (Líška et al. 2016), sorghum (Bathoova et al. 2018), and rice (Hinrichs et al. 2017), compared to the dimorphic exodermis features alternating long and short cells (Clarkson et al. 1987; Peterson 1989) as developed in onion *(Allium cepa*) (Barrowclough et al. 2000) and Asparagus (*Asparagus officinalis*) (Hose et al. 2001). While the exodermis in Poaceae is typically uniform, its development can be constitutive or inducible, depending on the species.

An inducible exodermis is formed in response to specific environmental factors, though the process of exodermal induction is not always straightforward and remains only partially understood, especially whether environmental cues change the cell type identity or the cell differentiation trajectory. For example, barley (*Hordeum vulgare spp. vulgare*), is commonly considered a species without an exodermis in seminal roots (Scott 1928; Kreszies et al. 2019, 2020a, b), however an exodermis has been observed in its adventitious roots (Gierth et al. 1999; Lehmann et al. 2000; Shiono and Matsuura 2024). Interestingly, biotic stresses, such as inoculation with the fungus *Chaetomium globosum*, can also induce a suberized exodermis in barley seminal roots preventing the fungal invasion beyond the exodermal barrier (Reissinger et al. 2003). In wild barley (*Hordeum vulgare spp. spontaneum*), a particular cultivar induces suberized exodermis differentiation in the basal root zone in approximately 20% of its seminal roots under osmotic stress (Kreszies et al. 2020a, b). Seaside barley (*Hordeum marinum*), a species adapted to waterlogging, also has inducible suberized exodermis to prevent radial oxygen loss under hypoxic conditions (Kotula et al. 2017). Similarly, some wild barley accessions and wheat roots may induce suberized exodermis differentiation under drought stress (Kreszies et al. 2020a; Ouyang et al. 2020).

In contrast, the differentiation of exodermis in rice is one of the key strategies to cope with waterlogging conditions. In addition to forming lysigenous aerenchyma to facilitate gas transport into the root, rice also develop an exodermal barrier induced by abscisic acid to facilitate better internal oxygen movement, enhance oxygen access, reduce radial oxygen loss, and protect the plant from toxic substances like hydrogen sulphide and reduced iron under hypoxic or stagnant conditions (Colmer et al. 1998; Shiono et al. 2022; Lin et al. 2023). In a similar strategy, *Zea nicaraguensis*, a wild maize relative tolerant to waterlogging, as well as maize and wheat, enhance waterlogging tolerance through inducing exodermal differentiation in response to hypoxic conditions (Enstone et al. 2002; Abiko et al. 2012). Unlike in rice, this exodermal differentiation does not form a true barrier to radial oxygen loss due to its distinct microstructure, which lacks glycerol esters in the exodermis (Jiménez et al. 2024).

However, even in species with a constitutive exodermis, as in maize, environmental factors can affect the differentiation of the exodermis. For example, in maize, exposure to low temperatures can accelerate the differentiation of the exodermis (Clarkson et al. 1987) and water availability fluctuations can enhance the deposition of lignin and suberin lamellae in the exodermis (Enstone and Peterson 1998). In addition, maize grown under low potassium and nitrogen enhances exodermal suberization (Vestenaa et al. 2024; Shiono et al. 2024). It is hypothesized that these enhanced or induced exodermal barriers help prevent the uncontrolled loss of nutrients into the surrounding soil or growth medium.

Despite its critical role in root function, the exodermis remains an underexplored aspect of Poaceae, particularly in comparison to the endodermis. The ability of the exodermis to regulate water, solute, and gas transport, as well as its inducibility under various environmental stresses, highlights its importance for plant adaptation to challenging growth conditions. Understanding the interplay between the exodermis and other root tissues, such as the endodermis, will be essential in developing crops with enhanced resilience to water stress, nutrient deficiencies, biotic stress, and toxic soil conditions.

### Sclerenchyma

Building on the protective and regulatory roles of the exodermis in root function, another key adaptation in the outer root layers of Poaceae is the development of specialized sclerenchymatous tissue. Cortical sclerenchyma- from the Greek Skleros, hard, and enkymos, infused, are just that; cortical parenchyma cells that have become toughened through deposition of secondary thickening compounds in their cell wall. This cell layer is defined based on the presence of uniformly deposited lignin in cell walls (Evert 2006; Jarvis 2012) which can occur most commonly in the hypodermis, but can also occur in the mesodermis as observed in perennial ryegrass and Cat grass (*Dactylis glomerata*) which develop a sclerenchyma ring (one to two layers) adjacent to the endodermis (Soper 1959). Sclerenchyma tissue is primarily composed of two cell types: fibres and sclereids, both characterized by thick, lignified cell walls that offer resistance to mechanical stress. Sclerenchyma fibres are typically elongated, with lignin deposition providing increased stiffness, while sclereids are more irregular in shape and contribute to the mechanical protection of the root. Sclerenchyma tissue in roots plays a critical role in providing structural support and rigidity, contributing to the overall mechanical strength of the plant, pest resistance, and as a barrier for radial oxygen loss (Cantó-Pastor et al. 2025).

Several Poaceae species develop sclerenchyma in the outer cortex (Fig. 4). Brachypodium forms a multilayered ring of sclerenchyma in the hypodermis that improves resistance to lodging (McCahill et al. 2024). Rice can develop several types of multilayered sclerenchyma in the hypodermis (Kondo et al. 2000) which can be enhanced by silicon application (Fleck et al. 2011) and may function as a radial oxygen loss barrier (Ejiri et al. 2020). Sclerenchyma have also been shown to be an important adaptation to resistance to the pest rice water weevil (*Orzophagus oryzae*) (de Bastos Pazini et al. 2022). Interestingly, endophytic bacteria isolated from mangrove plants, when applied in rice, were able to trigger the development of sclerenchyma, and by this improve rice growth under hypoxic conditions (Alghamdy et al. 2024). This, similarly to microbial-induced suberization, exemplifies how plant-associated microbes promote cortex differentiation protecting the plant from stress. A type of cortical sclerenchyma, multiseriate cortical sclerenchyma, which may improve root penetration ability in compacted soils is discussed below.

#### Multiseriate cortical sclerenchyma

Multiseriate cortical sclerenchyma is a type of sclerenchyma formed in the outer cortex. The key difference between multiseriate cortical sclerenchyma and sclerenchyma (described above) are the size and position of sclerenchymatous cortical cells (Figs. 3 and 4). Multiseriate cortical sclerenchyma consists of multiple layers of small cells with thick, deeply lignin-encrusted cell walls located in the outer cortex of nodal roots and has been described in the roots of many Poaceae, including maize, sorghum, barley, and wheat (Schneider et al. 2021). Developmentally, multiseriate cortical sclerenchyma forms primarily in younger nodal roots of axial roots and persists throughout plant maturity, but it is absent in seminal and lateral roots. In maize, multiseriate cortical sclerenchyma is observed after the zone of differentiation and lateral root formation in axial nodal roots. The thickness of cortical cell walls within multiseriate cortical sclerenchyma remains unchanged once it has developed (Schneider 2022). Multiseriate cortical sclerenchyma, influencing the biochemical composition and cell wall thickness of cortical cells, has the potential to alter the apoplastic and symplastic pathways through which water and solutes travel, however it is unknown if multiseriate cortical sclerenchyma functions as an apoplastic barrier (Schneider 2022).

Beyond its potential function as an apoplastic barrier, multiseriate cortical sclerenchyma enhances root mechanical properties, increasing tensile strength enabling better penetration in mechanically impeded soils (Schneider et al. 2021). Maize with multiseriate cortical sclerenchyma had greater root depth and plant performance in compacted soils in the field (Schneider et al. 2021). Similarly, smaller outer cortical cells in maize improve root tensile strength, bending force (Chimungu et al. 2014), and plant growth in dry, hard soils (Klein et al. 2020). Maize roots also may develop multiseriate cortical sclerenchyma under salt or osmotic stress (Shen et al. 2015). Multiseriate cortical sclerenchyma has also been proposed to influence water and nutrient uptake, resistance to pests, symbiotic interactions, radial water loss, microbial interactions, and soil carbon deposition (Schneider 2022).

The genetics of sclerenchyma formation in the root cortex of Poaceae is still not fully understood, but recent studies have begun to shed light on key regulatory genes and pathways involved. Currently, no molecular markers for sclerenchyma exist. Compared to the extensive research on sclerenchyma development in stems and nodes, root cortical sclerenchyma has received less attention. *SECONDARY WALL NAC7* has been identified as a putative regulator of mechanically responsive cortex cell wall development at the root base in Brachypodium (McCahill et al. 2024). Genome-wide association analysis and quantitative trait loci mapping in maize identified multiseriate cortical sclerenchyma as being linked to a genetic marker that encodes a MEI2-like RNA-binding protein (Schneider et al. 2021). In rice stems, *BRITTLE CULM 5 (BRITTLE NODE)* plays a role in secondary cell wall formation in sclerenchyma tissue of nodes (Aohara et al. 2009) and a knock-out of transcription factor WRKY53 thickens sclerenchyma cell walls surrounding root vasculature (Xie et al. 2021).

The connection between cell wall thickenings (e.g. endodermis, sclerenchyma, exodermis) and environmental responses is well established, yet the precise mechanisms governing this relationship remain poorly understood. Environmental stresses can trigger modifications in cell wall morphology, structure, or chemical composition within a single cell type, facilitating an adaptive response to specific challenges. The presence of multiseriate cortical sclerenchyma is the result of a localised cell thickening, though may be preceded by the proliferation of cortical cell layers with the potential for these cell wall modifications, and warrants further exploration. These changes may reinforce mechanical strength, biotic stress tolerance, regulate water movement, or influence interactions with microbial communities. However, it remains unclear how multiple concurrent stresses influence these modifications, whether they act synergistically, antagonistically, or in an entirely novel manner.

In species known for their resilience to particular abiotic or biotic stressors, characteristic patterns of cell wall thickening may play a key role in their survival. Identifying such patterns and understanding their functional significance could provide valuable insights into stress adaptation mechanisms. If certain modes of cell wall reinforcement and barriers (i.e. location and composition) prove to be broadly beneficial, introducing these traits into more sensitive plant species or genotypes through breeding or genetic engineering may enhance their ability to withstand environmental challenges. This approach could open new avenues for improving crop resilience and sustainability in increasingly unpredictable climatic conditions.

## Discussion

### Importance of root cortical tissues for global agriculture

Root traits are central to soil resource uptake, and a deeper understanding of their genetic regulation and ecological significance is crucial. Harnessing the potential of the root cortex to enhance resource use efficiency represents a critical strategy for developing crops and cropping systems that can support a growing global population in a changing climate. A deeper understanding of how the root cortex of Poaceae influences resource capture is essential, as water and nutrient availability are major constraints on plant growth across most terrestrial ecosystems. In natural systems, exploring the role of the cortex in root function will provide insight into key factors driving ecosystem productivity and resilience, with implications for mitigating the impacts of climate change and human activity. In agricultural systems, optimizing cortical tissues offers a promising avenue for sustaining crop yields despite soil degradation, increasing population pressure, and environmental challenges. Intensive fertilization and irrigation in high-input systems are costly, environmentally damaging, and unsustainable, while in low-input systems, limited soil fertility and drought severely restrict productivity, food security, and economic stability. These pressures are intensifying due to climate change, freshwater depletion, and soil degradation. Investigating the fitness landscape of root cortical tissues may facilitate the breeding of crop varieties with enhanced capacity for soil resource acquisition, ultimately improving agricultural sustainability and resilience in the face of global change.

### Breeding challenges

While there is an urgent need to incorporate root traits into plant breeding and selection programs, a few key challenges remain. Phenotyping methods to image and annotate cortical tissues have been developed (Strock et al. 2019, 2022; Sidhu and Schneider 2024a, b), although not yet available at the throughput needed for breeding. Genetic mapping studies have demonstrated that root cortical tissues are heritable, though they are typically highly quantitative, controlled by many genes with small individual effects (Schneider et al. 2020a). Compared to above-ground traits, relatively few genetic loci have been identified for root cortical tissues. However, several quantitative trait loci have been mapped including root cortical aerenchyma in *Zea* species (Mano et al. 2005, 2007), multiseriate cortical sclerenchyma in maize (Schneider et al. 2021), and areas of the cortex, cortical cells, and root cortical aerenchyma (Burton et al. 2013; Schneider et al. 2020a). Recent research has also demonstrated that plasticity in root anatomical tissues is genetically regulated, with different genetic loci influencing plastic responses of the cortex compared to those controlling cortical tissues under optimal conditions (Schneider et al. 2020a).

This polygenic nature of the genetic control of cortical tissues presents a challenge for traditional breeding approaches focused on single-trait selection. Implementing an ideotype breeding strategy, which requires stacking hundreds of genes, may not be feasible using conventional methods. However, modern techniques such as genomic selection offer a valuable tool for simultaneously selecting multiple loci. These approaches should account not only for individual root tissues but also for integrated phenotypes. Additionally, incorporating wild germplasm and landraces into genomic selection training sets is crucial, as elite germplasm has primarily been developed under high-input conditions, potentially selecting against root plasticity and foraging strategies advantageous in low-input environments. Wild relatives and landraces likely harbour greater variation and plasticity for root traits, including cortical tissues, compared to elite crop varieties, making them valuable genetic resources.

### Breeding and domestication have altered cortical tissues of Poaceae

The root cortex has undergone significant evolutionary changes from early vascular plants to modern root systems. In the rhizoid-based rooting systems of early vascular plants, the rhizoid-bearing axes closely resembled above ground stems, with minimal structural differences and some degree of developmental interchangeability (Cronk et al. 2002; Kenrick and Strullu-Derrien 2014). These axes featured distinct tissue layers, including an outer epidermis (with stomata in some species), a supportive hypodermis, a cortex, and a central vascular cylinder (Kerp 2001; Edwards 2003). In many Rhynie Chert (a siliceous sedimentary rock formation, located near Rhynie, Scotland, known for containing an abundance of exceptionally high-quality early plant fossils) plants, the prostrate rhizomatous axes displayed a basic tissue patterning similar to aerial stems, further highlighting the early structural continuity between rooting and shoot systems. Over time, the cortex evolved specialized functions, such as regulating nutrient and water transport, providing structural support, and interacting with soil microorganisms, ultimately contributing to the diverse and efficient root architectures and tissues seen in modern plants.

Although research on the effects of breeding and domestication on root cortical tissues in Poaceae remains limited, existing studies suggest that these processes have significantly influenced cortical anatomy. Domestication has indirectly shaped cortical tissue traits, likely due to selection for overall plant performance, yield, and adaptation to changing agricultural environments. Variation in cortical tissues, including cortical area and aerenchyma area, was assessed across 256 accessions in the *Zea* genus, including maize landraces, teosinte *parviglumis* (*Zea mays subspecies parviglumis)*, teosinte *mexicana* (*Zea mays subspecies mexicana*), teosinte *huehuetenangensis* (*Zea mays subspecies huehuetenangensis*), teosinte *perennis* (*Zea perennis*), teosinte *luxurians* (*Zea luxurians*) and teosinte *nicaraguensis* (*Zea nicaraguensis*). In general, teosintes had a reduced total cortical area, cortical cell size, and aerenchyma area compared to landraces (Burton et al. 2013). In an analysis of germplasm developed more recently, sixteen commercially successful maize varieties over the past century, root cortical aerenchyma had no change across this time period, however cortical cell size increased by approximately 5% in the most modern lines compared to older germplasm (York et al. 2015). These findings suggest that the change in size of cortical tissues, including increased cortical cell size and total cortical area, has been a gradual and continuous process from teosintes to modern maize, likely reflecting changes in plant size and adaptations to changing agronomic practices and soil resource availability.

Changes in the proportion of aerenchyma tissue in the cortex have also been observed between teosintes and modern maize. Teosinte *parviglumis* and teosinte *mexicana* have a reduced aerenchyma percentage compared to modern maize (teosinte *parviglumis* cortex is around 6.7% aerenchyma tissue, teosinte *mexicana* cortex is around 6.2% aerenchyma tissue, modern maize cortex is around 16.6% aerenchyma tissue) (Burton et al. 2013; Schneider et al. 2020a; Lopez-Valdivia et al. 2022). However, the cortex of teosinte *nicaraguensis* is composed of around 23% aerenchyma tissue under optimal conditions. This proportion was the second largest in a group of 256 *Zea* accessions (Burton et al. 2013). In a separate study evaluating teosinte *nicaraguensis* under waterlogged conditions, Abiko et al. (2012) observed cortical tissues to contain (proportionally) 30% aerenchyma. The development of more aerenchyma tissues under waterlogging combined with increased lignin deposition in the outer cortical cells of teosinte *nicaraguensis* acted as a barrier which prevented oxygen loss and improved plant performance under deoxygenated nutrient solution (Abiko et al. 2012). Since this combination of cortical tissues is present in teosinte *nicaraguensis*, but not in parviglumis or mexicana, it appears to be a local adaptation to the high precipitation rates of Nicaragua (the origin of teosinte *nicaraguensis*), rather than a trait associated with maize domestication.

Changes in other cortical tissues have also been observed among teosintes, landraces, and modern germplasm. Multiseriate cortical sclerenchyma is found in some modern maize inbreds but not in teosinte *parviglumis* and teosinte *mexicana*. This suggests multiseriate cortical sclerenchyma may represent an adaptive trait acquired during domestication (Schneider et al. 2021). Similarly, multiseriate cortical sclerenchyma has not been observed in landraces of wheat or barley, but is present in many modern cultivars (Schneider et al. 2021). Evidence suggests that crop domestication involved selecting traits that enhance adaptation to challenging soil environments (Lynch et al. 2021). Although absent in wild ancestors of cereals, multiseriate cortical sclerenchyma has been documented in cultivated lines, including 5,280-year-old maize roots from Tehuacan Valley in central Mexico (Lopez-Valdivia et al. 2022). Interestingly, the earliest evidence of multiseriate cortical sclerenchyma coincides with the advent of irrigation in the Tehuacan Valley, where ancient channels reveal the implementation of irrigation systems around 6,000 years before present (Neely et al. 2022). Simulations of ancient soils suggest that this period also marks a shift in nitrogen availability from shallow to deeper soil profiles, likely driven by increased leaching associated with irrigation (Lopez-Valdivia et al. 2025). Simulations of ancient maize root systems and their corresponding soil environments suggest that the emergence of multiseriate cortical sclerenchyma, would have improved the root depth and plant performance under these conditions (Lopez-Valdivia et al. 2025). This adaptation likely allowed maize to access deeper water and nitrogen resources, contributing to its success in the Neolithic agricultural landscape of the Tehuacan Valley.

As discussed in the exodermis section, seminal roots of modern barley are commonly considered absent of exodermis, however wild barley from Jordan (arid to semi-arid climate) exposed to osmotic stress increased the deposition of suberin in the endodermis and exodermis, creating apoplastic seal that forced the symplastic water uptake, and therefore ensuring water uptake and avoiding water loss (Kotula et al. 2017; Kreszies et al. 2020a). This suggests that the exodermis, though often absent in some cultivated varieties, remains a key adaptive trait in wild barley, allowing plants to cope with drought stress and biotic stresses. Such findings highlight how domestication has, in some cases, led to the loss or reduction of stress-adaptive root traits, potentially making modern crops more vulnerable to environmental fluctuations.

Despite the clear impact of domestication and breeding on cortical tissues, it remains uncertain whether these traits arose through direct selection. To our knowledge, no breeding programs have explicitly targeted root cortical traits for improvement. Instead, the observed changes may have resulted from indirect selection, as root architecture and tissue composition influence water and nutrient uptake, which in turn affect overall plant productivity and yield. Moving forward, direct selection for beneficial cortical traits, such as optimized cortical area, aerenchyma formation, exodermis differentiation, or sclerenchyma development, could present new opportunities for enhancing crop performance under diverse environmental conditions. However, future breeding strategies must be guided by a mechanistic framework that considers how root and shoot traits interact to shape resource acquisition, stress tolerance, and overall plant fitness. Understanding these complex relationships could help unlock new pathways for improving crop resilience and sustainability in a rapidly changing agricultural landscape.

### The cortex in context: future perspectives and research opportunities

The Poaceae root displays a range of structural and functional diversity, which can be seen in the organization of its cortical tissue. The dynamic nature and diverse developmental pathways of cortical parenchyma cells result in a variety of tissues developed within and between the endodermis and hypodermis. The structure and function of tissues in the root cortex gives insight into leveraging the potential of the cortex for the development of more productive crops in a range of environments. Although significant strides have been made in recent years to characterize cortical tissues and their function in Poaceae, many questions remain unanswered. Understanding the developmental plasticity and the structural and functional variations of these tissues will provide insights into how plants adapt to their environment, allocate resources, and respond to stress. Here, we discuss several research opportunities in order to facilitate our understanding of this composite layer of tissue(s).

#### Cortical tissues influence root exudation and microbiome recruitment

Variation in cortical tissues may translate into diversity of niches for microbial associations in the rhizosphere, rhizoplane, and cortex (Galindo-Castañeda et al. 2022). Several root cortical tissues, including root cortical aerenchyma and the presence of root cortical senescence alter oxygen and water content, and presumably nutrient availability, in the rhizosphere. This too can influence carbon rhizodeposition in determining the structural and physical characteristics of niches and microhabitats for microbial communities (Galindo-Castañeda et al. 2022). For example, root cortical aerenchyma can provide avenues for gas diffusion, providing aeration to the root tip and rhizosphere, and allow for expulsion of gases such as carbon dioxide, ethylene, and methane from the root and surrounding soil (Shannon et al. 1996; Colmer 2003). This can serve to mitigate the adverse effects of anoxic soil conditions on roots and fulfils the oxygen requirements of soil microorganisms, which would otherwise compete with the root for available oxygen.

Root exudation benefits plants by stimulating beneficial microbes and promoting nutrient acquisition (Mommer et al. 2016). How root anatomy, and therefore the biochemical composition and thickness of the root (particularly the outermost cortical layer) influences root exudates along root systems, across different root classes, and in different species remains an open question. Presumably, outer cortical tissues (e.g. hypodermis, exodermis, sclerenchyma, multiseriate cortical sclerenchyma) represent major control points for root exudation and, when lignified or suberized, act as a barrier for the release of root exudates that diffuse through the apoplastic spaces (Canarini et al. 2019). Cortical tissues influencing the biochemical composition of cell walls, number of cortical layers, and apoplastic barriers have the potential to influence root exudation and the microbiome by altering the apoplastic space and the length of the symplastic pathways through which exudates travel (Galindo-Castañeda et al. 2022). Arabidopsis plants with altered apoplastic barriers (by mutation resulting in defective Casparian strip or endodermal suberization) show changes in their general exudation profile (Salas-González et al. 2021; Durr et al. 2021). Different root cortical tissues may support a specific composition of root exudation, area of microbial colonization or habitat within the host root (Galindo-Castañeda et al. 2018, 2022). For example, several important metabolites including hormones and exudates are synthesized in the root cortex including lignin precursors, auxins, and abscisic acid (citations) and the development of root cortical aerenchyma and root cortical senescence may significantly reduce living cortical parenchyma for synthesis. The link between spatiotemporal variation in cortical tissues with other phenotypes including exudates, carbon rhizodeposition, and metabolite concentration is poorly understood but crucial for understanding root-microbial associations (Galindo-Castañeda et al. 2022).

#### Relationships between axial and lateral root cortical tissue

In many Poaceae, lateral roots comprise the majority of root length and surface area and play a large role in nutrient and water uptake (Schneider et al. 2017a, 2020b). However, the structural and functional characterization of lateral roots in many species is rare, nevertheless may have important functions in the plant. For example, in rice, lateral roots have been described as three distinct classes, which has been mirrored in pearl millet and maize based on growth rate and anatomy (Passot et al. 2016, 2018; Affortit et al. 2024). In rice these different lateral root types have different functional contributions to plant nutrition and water acquisition (Watanabe et al. 2020). Further subcategories have been described according to cortical cell wall properties. For example, thick lateral roots (also known as L(large)-type) have a similar anatomical structure to nodal roots, including an exodermis, sclerenchyma, aerenchyma, and endodermis. However, thin lateral roots (also known as S(small)-type) have no aerenchyma formation and fewer files of cortical cells when compared to the axial root (Kawata et al. 1977; Yano et al. 1996). While the variation of anatomy of lateral roots has been studied extensively in rice, to date it has not been fully explored in other Poaceae species.

Another open area of research is the relationship between axial and lateral root cortical anatomy. For example, lateral roots that emerge from an axial parent root often do not express correlated anatomical features and often lateral roots display more simple cortical phenotypes (i.e. commonly lack of deposition of secondary metabolites). In addition, root cortical senescence and the formation of multiseriate root cortical sclerenchyma (Schneider et al. 2017b, 2021) are just a few examples of tissues that are rare or absent in lateral roots, despite the parental axial root having a strong phenotype. The capacity for lateral roots in Poaceae to alter their cortical phenotypes spatially and temporally, the relationship of lateral root cortical phenotypes with the axial parent root, and the genetic control of lateral root cortical phenotypes are important open research questions.

Building on the anatomical diversity of lateral roots, their development and emergence involve complex interactions with surrounding cortical and epidermal tissues, highlighting the dynamic remodelling required to facilitate their growth and integration into the root system. Due to physical processes of lateral root emergence and mechanical stability of more mature lateral roots, cortical tissues in the axial root are significantly altered around the base of lateral roots. In many grasses, lateral root primordia develop from the pericycle, the cell layer located just inside the endodermis. As the primordia grow, they must navigate through overlying cortical and epidermal tissues to emerge and interact with the soil (Péret et al. 2009). This process requires precise regulation to ensure emergence without excessive damage to the parent root. Lateral root development and emergence has been well studied in Arabidopsis. For example, auxin-induced cell wall loosening permits outer layer separation during primordia expansion. Before lateral root initiation, pericycle cells swell, requiring auxin signalling in the overlaying endodermis to accommodate (Vermeer and Geldner 2015). Although much progress has been made in Arabidopsis, studies in Poaceae are limited. In maize and other cereals, the endodermis plays an active role in lateral root development and lateral root caps and epidermal layers originate from the endodermis (Orman-Ligeza et al. 2013). Histological studies in maize suggest that endodermal cell wall lignin degrades during lateral root formation (Karas and McCully 1973), but the mechanism of lateral roots passing through the endodermis and exodermis during emergence remains unknown.

Not only does lateral root emergence impact cortical tissues, but also after emergence, parent root cortical anatomy is altered around the base of lateral roots, presumably to maintain structural support of the lateral root. For example, there is reduced or delayed root cortical aerenchyma and root cortical senescence formation around the base of lateral roots (Henry and Deacon 1981; Justin and Armstrong 1987; Evans 2004). Therefore, the number and distribution of lateral roots may have implications for axial cortical tissues and their function and utility in stressful environments.

#### Ephemerality in root tissues

Ephemerality, the tendency of something to be short lived, is often seen in plants, especially during flowering and reproductive processes. Once the purpose of a structure has been achieved, it is determined not to be able to complete its function, or it reaches the end of its utility, senescence and or reallocation of resources can take place. However, the governance and function of senescence and ephemerality in root tissue is less clear than in shoot tissues. Beyond the root cortex, we feel it is poignant to highlight the other well-noted occurrences of short-lived structures in the root; we consider this ephemerality to fall in concert with the phenomenon of root cortical senescence described previously.

**Fig. 5:**
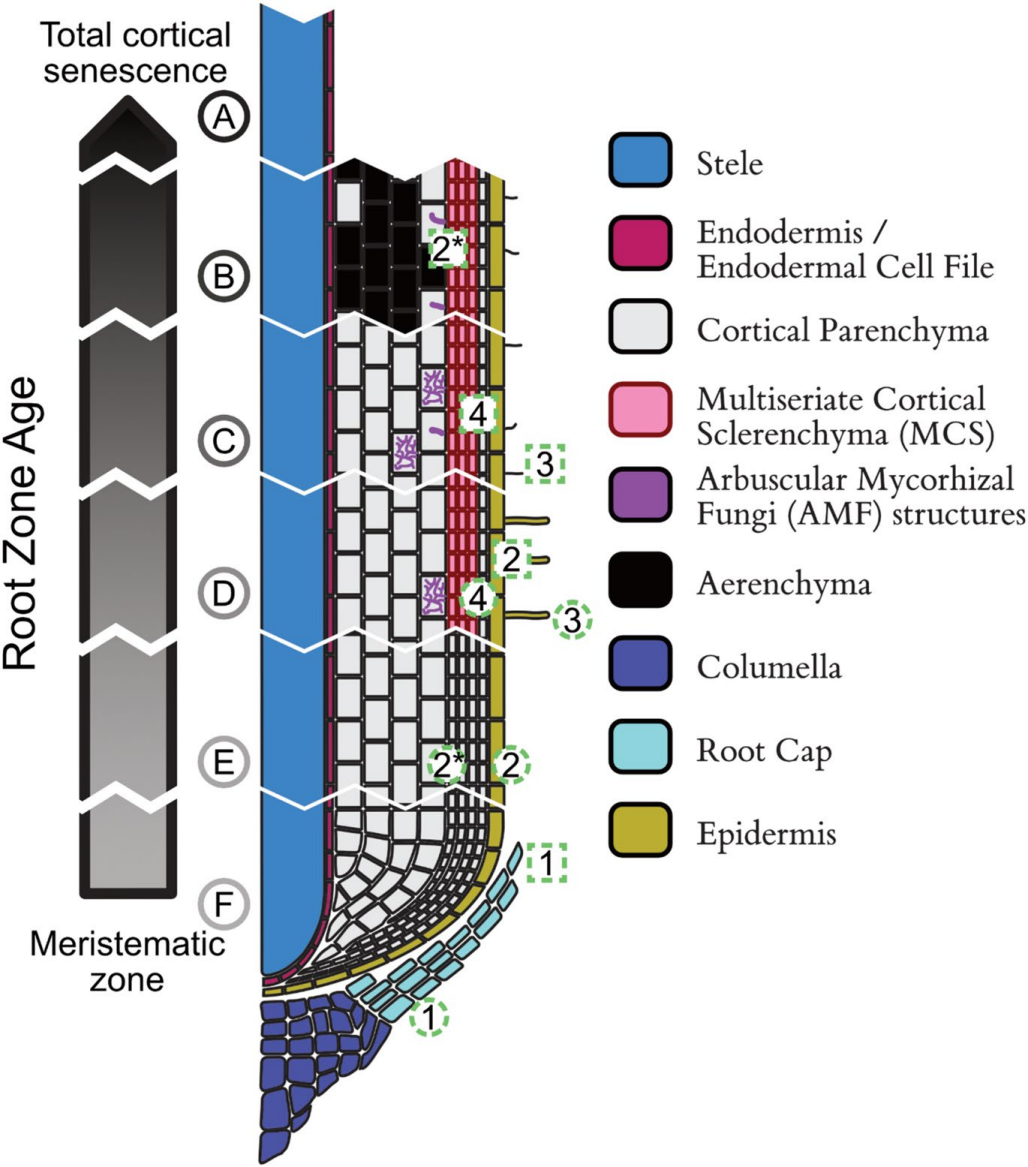
Ephemeral root structures. Temporal development and transformation of anatomical structures of the Poaceae roots. Numbers represent different anatomical structures: 1) root cap, 2) cortical parenchyma, 3) root hairs, and 4) arbuscular mycorrhiza. Circles represent the first appearance of the structure and squares represent their transformation/disappearance. Asterisk (*) indicates different fates. For instance, cortical parenchyma could transform into either multiseriate cortical sclerenchyma or aerenchyma. Letters represent different tissue ages

Arbuscules formed in cortical parenchyma cells by arbuscular mycorrhizal fungi, structures such as root hairs, as well as lateral root cap cells, all exhibit a degree of ephemerality that mirrors the phenomenon of root cortical senescence (Figs. 4 and 5). In each case, the temporary nature of these structures is tied to specific functional roles that ensure the plant's growth and adaptation to its environment. As such, understanding these transient components can offer deeper insights into the broader process of root maturation, resource acquisition, and plant resilience.

Arbuscular mycorrhizal fungi are a category of symbiotic fungi that are distinguished by their formation of arbuscules (highly branched nutrient exchange structures) within root cortical cells. This symbiosis can be a significant contributor to plant performance, and in maize, has been shown to contribute approximately one-third of grain yield increase (Ramírez-Flores et al. 2020; Grondin et al. 2024). Once the fungal hyphae are in the root, arbuscules are formed inside cortical cells. The hyphae branches, puncturing the plant cell wall, and once inside, the plant cell membrane is extensively remodelled to envelop the highly branched arbuscule, providing a large surface area for nutrient exchange in the form of the peri-arbuscular membrane (Luginbuehl and Oldroyd 2017). Considering the importance of this symbiosis to the plant, the potential (relatively longer) longevity of root cortical cells compared to arbuscules, and the metabolic cost involved in the formation of the peri-arbuscular membrane, it is somewhat surprising that an individual arbuscule in a cortical cell may last as little as 48 h from its formation and collapse (McGaley et al. 2025). While it is clear that this is a tightly regulated process, and the cortical cell remains alive (and capable of re-colonization) after the event, the reason for this rapid arbuscule turnover is less clear (Luginbuehl and Oldroyd 2017). It is suggested that this enables tight regulation of the symbiosis within the plant. This early termination of the arbuscule may contribute to the individual host cells’ longevity. Arbuscules’ high turnover may serve a similar function to the turnover of root hairs and other cell death processes in the root; by reducing metabolic expenditure on maintaining a matured root zone, resources may be better spent on producing new roots or supporting exploration of the soil in new areas. Perhaps too, this turnover serves the function of encouraging fungus to increase root exploration forward along the longitudinal root axis, rather than supporting the more sedentary production of vegetative (vesicles) and reproductive (spores) structures.

**Fig. 6:**
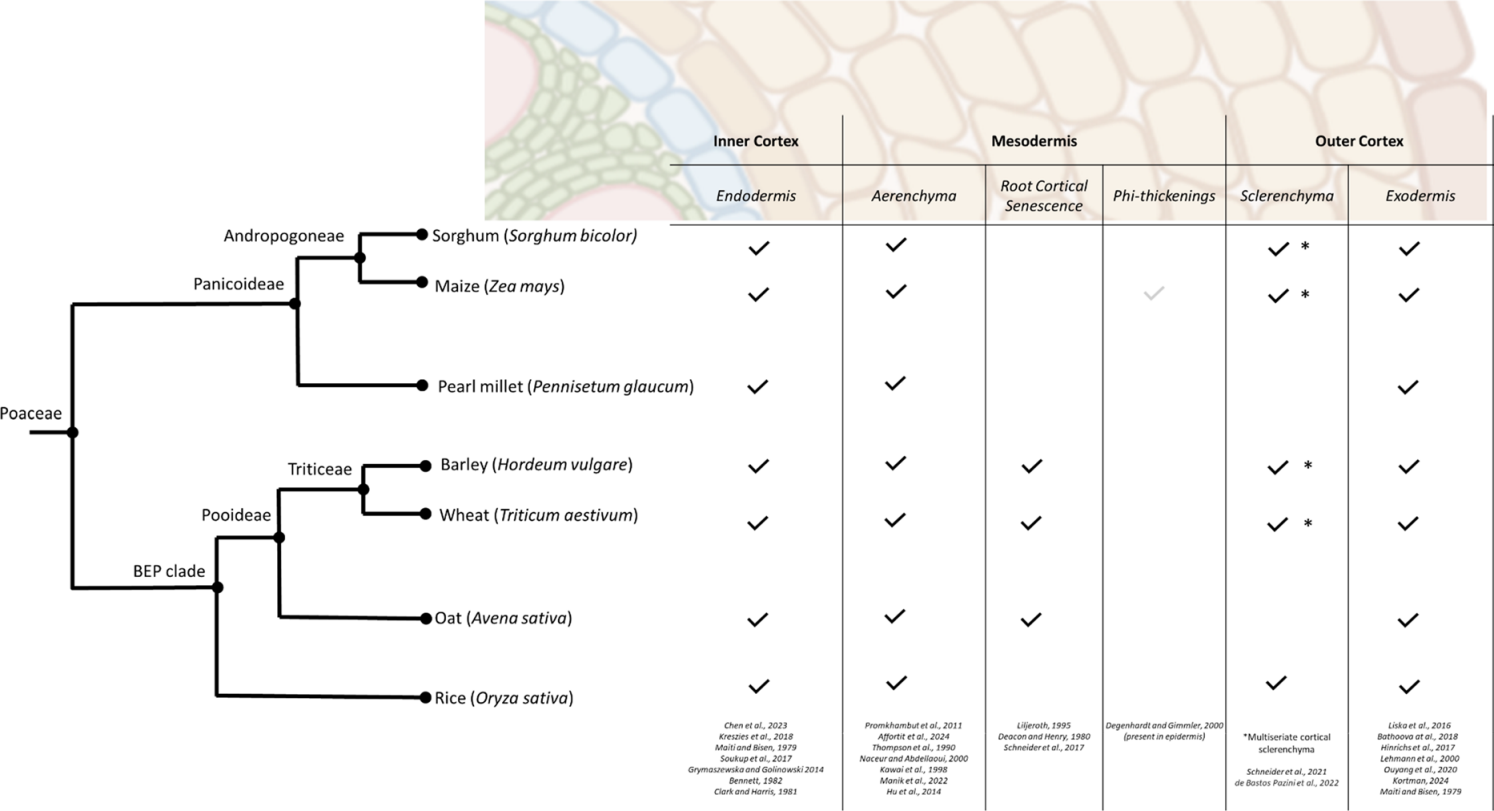
Cladogram of several crop species in the Poaceae family. Cortical tissues that have been documented in these species are marked. Not every species in the Poaceae family has the potential to develop all cortical tissues described above. Tissues marked can be formed simultaneously or in succession; constitutively or inducibly. Phi-thickenings in maize are marked in grey, as these have not been observed in the cortex, but epidermis

Root hairs are perhaps one of the most prominent short-lived structures in the root. Root hairs are narrow epidermal structures of cylindrical tip growth driven extrusions from the epidermis, which greatly contribute to increasing the surface area of the root for nutrient uptake. Despite the great utility of root hairs, their ephemerality is well documented across many species, typically only lasting a few days to a week from genesis to senescence (Figs. 3, 4, 5, and 6). This rapid turnaround can be attributed to maximising the efficacy of the root’s dynamic growth pattern; with the continuous growth of roots into unexplored soil regions, continual production of new root hairs to maximise exploration, and attrition of older root hairs to reduce metabolic cost is an expedient strategy (Bates and Lynch 2001) Moreover, root hairs can be responsive to environmental stimuli, such as nutrient availability and soil conditions, further reinforcing their transient role in resource acquisition(Fusseder 1987; Dolan 2017; Saengwilai et al. 2021; Fan et al. 2022). As part of root cortical senescence, the epidermis is also lost (Lascaris and Deacon 1991). It is unclear how the vitality of different cell types and tissues interact in the root, however it would be sensical and advantageous for there to be coordination between the timings of senescence between these tissues, as opposed to the underlying tissue to the epidermis and root hairs becoming non-functional before the root hairs have achieved maximal nutrient extraction. Understanding the regulation and signalling implications of root hair senescence may shed light on the initiation of cortical senescence.

Short-lived cells are also present at the root tip. The root cap encloses and safeguards the meristematic stem cells at the actively growing root tip. Unlike the root epidermis, which extends along the entire root, the root cap is confined to the meristematic zone. As a result, while epidermal cells persist beyond maturation, root cap cells must periodically shed to prevent excessive growth beyond the meristematic region (Fig. 5-F1). This continuous renewal of root cap cells is advantageous, as these cells endure mechanical stress while the root penetrates the soil. Additionally, this dynamic turnover is essential for maintaining the organ's size and positioning at the root tip (Kumpf and Nowack 2015; Di Mambro et al. 2019). Due to the rigid nature of plant cell walls, which prevent cell migration, root cap stem cells must consistently produce new cells in coordination with root stem cells. Different plant species have evolved distinct mechanisms to regulate this process. In Poaceae, root cap cells typically lose their cell wall attachments and are released as individual, viable ‘border cells’ into the rhizosphere (Driouich et al. 2007). The number of these detached border cells varies, ranging from a few dozen to tens of thousands per root tip, depending on species and root age (Hamamoto et al. 2006). Though important for root tip growth through the soil, this is a metabolically and photo-assimilate costly phenomena, and unlike cortical senescence, doesn’t give the opportunity for resource remobilization (Driouich et al. 2007). High-cost activities such as the shedding of root cap cells during tip growth are one of the processes that may benefit from the reduced cost of mature tissue resulting from cortical senescence and may share regulatory pathways with root cap cell turnover in ensuring that the timing and location of this cell loss is well coordinated for maximum utility. The shedding of lateral root cap cells alters the rhizosphere and the microbiome of the root, affecting what microbes the mature root may encounter (Hawes et al. 2016), and what symbiotic or antagonistic relationships the root cortex may later develop. These shed cells can also modulate the ion content of the rhizosphere (Hawes et al. 1998), further affecting the environment encountered by the mature cortex and its developmental trajectory.

The widespread occurrence of ephemeral structures in root systems, including cortical parenchyma (e.g. the development of root cortical senescence), root hairs, arbuscules, and the cells in the lateral root cap, underscores the dynamic nature of root development and function. These transient structures, while short-lived, play essential roles in nutrient acquisition, symbiotic interactions, and environmental adaptation. However, despite growing recognition of their importance, many fundamental questions remain unanswered. Future research should focus on elucidating the genetic and physiological regulation of these ephemeral structures, particularly how their turnover is coordinated with whole-plant resource allocation and stress adaptation. Characterizing the lifespan of different cellular structures in response to the local environments biotic and abiotic factors may reveal shared regulatory processes governing continuance or senescence of different structures, and in turn explain a portion of the anatomical heterogeneity observed along the root axis. Additionally, exploring the evolutionary drivers of root tissue ephemerality across diverse plant lineages could provide insights into how plants optimize growth strategies in different environments. A deeper understanding of these processes has the potential to inform crop breeding efforts aimed at enhancing root efficiency, stress resilience, and overall plant productivity in the face of changing environmental conditions.

#### Cortical tissues are species-dependent and variable in space and time

Not every species in the Poaceae family has the potential to develop all cortical tissues described above, nor necessarily to develop certain tissue combinations even when present individually (Fig. 6). For example, lysigenous root cortical aerenchyma and an exodermis commonly develop in rice and wheat, while, in rice root cortical senescence has not been observed (Schneider and Lynch 2018). This highlights various strategies different species in the Poaceae family undertake for stress tolerance and growth. These species-dependent strategies can also provide insight into the functional utility of many of these tissues as, presumably, these phenotypes were indirectly selected in native or breeding environments for stress tolerance and plant productivity (Fig. 6).

Subsequently, the relevance of model organisms in determining the function and genetic control of many of these tissues is limited. For example, Arabidopsis has a simple and highly regular root cortical structure that often only consists of two to three cortical layers (i.e. one layer of endodermis and one to two layers of cortical parenchyma) (Schneider and Lynch 2018; Kim et al. 2022; Yang et al. 2024) that do not provide the variation needed to understand the function and variability of most of these cortical tissues or the mechanisms governing the proliferation of cortical cell layers. For example, aerenchyma, multiseriate cortical sclerenchyma, and root cortical senescence have not been observed in Arabidopsis. While model organisms may not present diversity in the cortex to study a wide range of cortical tissues, important insights from the function and development of a relatively simple cortex in Arabidopsis can provide key insights into the potential structure and function of a few cortical tissues in Poaceae, including the endodermis and cortical parenchyma. For example, endodermal development involves two differentiation steps, the deposition of lignin and suberin, the latter presumably switching the function from an absorbing to a protecting cell type during plant development. This endodermal functional trajectory in the root cortex is especially notable when viewed alongside the gradated senescence of the epidermis, outer, and middle cortical cell layers. As a root region ages, root hairs senesce, aerenchyma can form, and the whole cortex may senesce; this leads to a reduction or halt in radial water transport, emphasizing the requirement of the endodermis as a protecting barrier. Hormones regulate suberization of the endodermis in response to environmental cues. For example, in Arabidopsis, abscisic acid can induce suberization and ethylene can degrade it. This degradation of endodermal suberization may play a large role in plant nutrient homeostasis (Barberon et al. 2016), but has not yet been studied or observed in Poaceae.

Because structure and function of the root cortex in many Poaceae species are spatially and temporally dynamic, this has limited our understanding of these tissues. For example, barley has the capacity to develop root cortical aerenchyma, root cortical senescence, exodermis, endodermis, and multiseriate cortical sclerenchyma. Nevertheless, it is unknown if barley genotypes have the capacity to develop these root phenotypes successively or simultaneously. For example, whether genotypes could develop root cortical aerenchyma or multiseriate cortical sclerenchyma early in growth, which subsequently develops into root cortical senescence remains an open question. If several of these cortical tissues have the potential to be developed alongside or following others, they could have the potential to have additive, synergistic, antagonistic, or even redundant functions for plant growth.

To make matters more complex, there is enormous genetic variation within a single species (and plant) for cortical tissues (Schneider et al. 2020a), and the expression of these tissues depends on the root type (e.g., shoot node origin), position along the root axis, tissue age (Schneider and Lynch 2018; Clément et al. 2022), and environmental factors (Schneider et al. 2020a). For example, in maize, root anatomical phenotypes vary by nodal origin of adventitious roots (Yang et al. 2019) e.g. multiseriate cortical sclerenchyma does not develop in older nodal roots (i.e. roots emerging from the most basal stem nodes) in maize (Schneider et al. 2021). This spatiotemporal variability in cortical tissues, in combination with the necessity for destructive phenotyping, makes it challenging to characterize changes in the cortex caused by ontogeny and environmental stress. However, it is critical to understand the utility and function of the timing and rate of development of root cortical tissues in different roots. For example, there are unexplored potential benefits and trade-offs of the rate of root cortical senescence formation. Presumably, a slower rate of root cortical senescence may allow for a greater capacity to remobilize nutrients and carbon from senesced cortical cells in other tissues before they are potentially lost to the rhizosphere. In contrast, an accelerated rate of root cortical senescence may result in a reduced nutrient and carbon demands of root maintenance improving stress tolerance. Characterizing the spatial and temporal changes in cortical features in response to stress and plant development and understanding their functional utility merits further research.

#### Root anatomy as a function of systemic plant development and constituent tissue development

Consideration of root tissue dependencies and their relationships with plant size is essential for understanding functional integration within the root system. The interactions between tissues can be synergistic, enhancing overall function, or antagonistic, where one tissue's development constraints another (Schneider 2022). Root anatomy exhibits interdependencies, driven by both allometric scaling (i.e. traits change proportionally with size) and hormonal signalling, so the size and developmental stage of the plant at the point of root development has a significant effect on anatomy. These relationships underscore the highly coordinated nature of root development, where structural features do not function in isolation but instead influence and constrain one another. In maize, for instance, cortical area and cortical cell file number scale proportionally with root diameter, and root diameter showed hyper-allometric relationships to shoot biomass (Yang et al. 2019). This suggests that root structural traits are not only developmentally linked, but also integrated with whole-plant growth dynamics, reflecting their importance in resource acquisition and transport.

These developmental interdependencies extend beyond overall root size and architecture to finer-scale tissue organization, where vascular patterning plays a crucial role in shaping cortical development. The arrangement of xylem and phloem poles appears to dictate spatial differentiation of tissues within the cortex. For example, in maize, lysigenous aerenchyma lacunae typically develop opposite of the phloem poles and living cortical parenchyma cell strands are positioned opposite of the protoxylem (Konings and Verschuren 1980). Similarly, in several species, endodermal and exodermal cells opposite phloem poles are typically more advanced in development than those opposite xylem poles (Konings and Verschuren 1980; Lux et al. 2004b) and cells adjacent to water-conducting xylem vessels remain as unsuberized passage cells (Andersen et al. 2018; Holbein et al. 2021; Kraska et al. 2024). The portion of the exodermis opposite of growing lateral root primordium often lacks suberin and lignin (Justin and Armstrong 1987; Armstrong et al. 2000; Soukup et al. 2002). These patterns suggest a developmental blueprint where vascular and cortical tissues co-develop in a way that may optimize hydraulic function and gas exchange.

Despite these known relationships, key uncertainties remain, particularly in how the vascular cylinder and surrounding tissues influence one another during cell elongation. The extent to which the endodermis constraints vascular cylinder expansion is unclear, yet it is likely that the thickness, number and width of endodermal cells before, and after cell elongation may be important factors in determining the vascular development potential, as well as the later hydraulic conductance of this cell layer.

Moreover, the development of these cortical tissues is dependent on shared hormonal signals. For example, ethylene, among other hormones, plays a central role in regulating the development of several cortical tissues in Poaceae, particularly in response to environmental stresses. One well-studied example is its involvement in the formation of lysigenous cortical aerenchyma, where ethylene accumulation triggers programmed cell death in cortical cells (Yamauchi et al. 2016). Ethylene also regulates root cortical senescence (Schneider et al. 2018) and plays a role in the development of multiseriate cortical sclerenchyma. In Arabidopsis, ethylene can also selectively degrade suberin in the endodermis (Barberon et al. 2016). Additionally, ethylene signalling interacts with other hormones, such as auxin and abscisic acid, to influence cortical cell differentiation and remodelling (Waadt et al. 2022). While increasing ethylene-related gene expression may enhance a beneficial trait under one set of conditions, it could also affect other traits in ways that may be maladaptive for a different environment. These findings underscore the pivotal role of ethylene in shaping cortical tissue development in Poaceae, particularly in response to environmental stress. This complexity highlights the importance of studying root anatomy as a dynamic system rather than a collection of independent traits.

#### Integration of aggregate root anatomical traits in understanding functional properties

As discussed above, the development of cortical tissues greatly affects root activity and function. However, tissues do not develop or function in isolation, so we must consider how these traits interact or co-function in the context of the root organ and the whole plant. The product of these interactions can be called compound traits, trait interactions, or integrated phenotypes, but all intend to describe instances of sympathetic co-occurrences of specific traits conferring specific functional properties. Often, integrated phenotypes contribute to root functions and plant performance. Thus, compound traits can be a useful conceptual framework for designing root ideotypes.

When evaluating root functional properties, we must consider the whole plant in context rather than individual traits pertaining to a single tissue. For example, in the cortex alone, radial hydraulic conductance presumably is dependent on the extent of secondary thickening of the exodermis and endodermis, the presence of multiseriate cortical sclerenchyma, the number of cortical parenchyma cell files, and the amount of aerenchyma. Functionally, the ratios between cortical tissue and vascular tissue, as well as that of aerenchyma to cortex (and xylem to stele), have been proposed as indices that reveal the adaptation of grasses drought and flooding stress, and can be used as targets to improve stress tolerance in crops. In a survey of 18 wild Poaceae adapted to contrasting soil water contents, dry (16% soil water content) adapted grasses had a significantly smaller cortex to stele ratio than wet (81% soil water content) adapted grasses, however, wet and dry adapted grasses both had significantly higher aerenchyma to cortex ratios than the middle (32% soil water content) adapted grasses (Yamauchi et al. 2020). This presents an interesting framework to understanding inter as well as intra specific adaptation of roots to abiotic stresses, and merits significant consideration of these trait ratios in further research.

On the other hand, while several tissues impact root functions, such as radial water transport, some of these cortical tissues may have redundant functions. For instance, both the exodermis and endodermis may regulate solute, water, and gas movement, suggesting potential functional redundancy. At the same time, synergistic relationships between anatomical and architectural traits have been identified, such as the interaction between root cortical senescence and lateral branching density (Postma and Lynch 2011; York et al. 2013; Schneider et al. 2017a). However, a systematic characterization of synergistic and antagonistic interactions between anatomical tissues is still lacking. One proposed synergy is the coupling of fewer cortical cell files with larger cortical cells to facilitate deeper rooting (Lynch 2013). Similarly, an integrated anatomical phenotype for drought tolerance has been suggested, comprising aerenchyma formation, larger cortical cells, and fewer cortical cell files (Klein et al. 2020). While an integration of root anatomical traits can go a long way towards explaining root radial water transport, to fully understand hydraulic conductance we must take the contribution of aquaporins (i.e. water channel proteins) into consideration. Aquaporins contribute significantly to root hydraulic conductance, and are differentially regulated under water stress (Grondin et al. 2016), meaning that hydraulic conductance can be different even within roots of similar anatomy. However, aquaporins have a more limited influence on the apoplastic pathway than cell to cell, so the relative importance of anatomy compared to aquaporin activity is context dependant (Bramley et al. 2007, 2009). In addition, programmed cell death of root cortical tissue (e.g. aerenchyma, cortical senescence) may influence the available living cortical area to host aquaporin proteins. These considerations highlight the importance of multiscale approaches in understanding root hydraulic conductance (Couvreur et al. 2018), and utilization of novel methods and systems approaches to facilitate this research (Heymans et al. 2021; Baca Cabrera et al. 2024). While these insights provide a foundation, a more comprehensive exploration of functional synergies and redundancies within cortical tissues is needed to better understand root adaptations to environmental constraints.

#### Trade-offs between cortical tissues and plant functions

The wide range of genetic variation in cortical tissues within and between species suggests that trade-offs in their functions and structures exist. These trade-offs could: 1) involve the development of one tissue constraining or preventing the development of a different tissue, 2) influence susceptibility to pests and diseases, and 3) affect radial nutrient and water transport, among many others. Loss of cortical parenchyma (e.g. root cortical aerenchyma) results in reduced habitat for arbuscular mycorrhizae fungi, potentially limiting symbiosis. In addition, the lignification of multiseriate cortical sclerenchyma may physically inhibit symbiosis. These effects have been observed in roots with cortical aerenchyma or sclerenchyma in the outer cortex, showing decreased arbuscular mycorrhizae colonization (Dreyer et al. 2010). However, this relationship appears to be complex as increased root cortical aerenchyma and reduced mycorrhizal colonization were correlated in inbred, but not in hybrid maize lines (Galindo-Castañeda et al. 2018; Strock et al. 2019). The relationship between many cortical tissues and colonization between symbionts is poorly understood and presumably involves many small anatomical and environmental nuances (Strock et al. 2019). From a physiological perspective, determining the relative contribution of the symbiosis compared to the root anatomical tissue itself for resource capture or plant growth would reveal the importance of the different mechanisms for plant productivity (Grondin et al. 2024).

The longevity of cortical cells and the deposition of secondary metabolites have important implications in determining the potential resistance of the cortex to a number of pests and diseases. After processes that reduce or destroy the root cortex, the cortex has a reduced capacity for defence (Yamauchi et al. 2020), while also potentially giving rise to a fuel source for rhizosphere microbial communities. For example, patterns of fungal invasion matched cortical cell death patterns during root cortical senescence (Deacon and Henry 1980; Henry and Deacon 1981; Deacon and Lewis 1986), and the rate of root cortical senescence strongly correlated with the amount of fungal biomass in the root (Liueroth et al. 1996). The colonization of *Fusarium verticillioides* was inversely correlated with living cortical area in maize inbred lines (Galindo-Castañeda et al. 2018). Presumably, cortical tissues affect the ability of pests and diseases to infest the root through the development of mechanical resistance (e.g. through the localized incorporation of lignin and/or suberin) (e.g., exodermal differentiation), reduced defence and production of defence compounds through the elimination of the cortex (e.g., root cortical senescence), or altered number of apoplastic avenues to spread throughout the cortex (e.g. root cortical aerenchyma) (Lynch et al. 2021).

Here, only a handful of potential trade-offs of cortical phenotypes are highlighted. There are many more potential trade-offs between tissues and the metabolic cost of the root, water and nutrient transport, infestation of pests and diseases, colonization of symbionts, and mechanical strength of the root. The utility of anatomical phenotypes is highly dependent on the environment, management factors, interaction with other root and plant phenotypes, and ontogeny and offers many research opportunities. It is critical to understand the fitness landscape of cortical tissues and their benefits and trade-offs for plant fitness in specific environments.

## Conclusions

The root cortex in Poaceae is a prime example of how evolutionary adaptations can drive the success of a plant family. The evolution of specialized structures and tissues and their respective functions in the cortex has helped grasses become one of the most widespread and ecologically important groups of plants on Earth. Their ability to thrive in diverse environments, from deserts to wetlands, highlights the remarkable plasticity and resilience of their root systems. This adaptability not only underscores their ecological dominance, but also their critical role in supporting human agriculture and ecosystem services.

A deeper understanding of the root cortex in Poaceae is essential for unravelling the mechanisms behind this adaptability, as its diverse tissues play a pivotal role in plant growth, resilience, and ecological success. Root cortex is a primary component of the root organ and in Poaceae plays an important and multifaceted role in plant growth and function. The diverse tissues and cell types present in the root cortex vary by species, genotype, and even root type and age are influenced by the environment and ontogeny. Cortical tissues have important roles for the uptake of water and nutrients, metabolic cost of root tissue construction and maintenance, gas exchange, disease and pest resistance, mechanical strength of the root, microbial associations, synthesis of metabolites, storage location for reserve materials, and subsequently stress tolerance and plant performance. We highlighted several important research areas and opportunities to better understand the root cortex and its function and potential for enhanced plant productivity. The influence of cortical tissues on plant growth and health are multifaceted over space and time and offer important research opportunities that have the potential to benefit basic research to applied breeding for crop improvement.
